# Canada’s 2025 AMR priority pathogens: Evidence-based ranking and public health implications

**DOI:** 10.1371/journal.pone.0330128

**Published:** 2025-09-17

**Authors:** Kahina Abdesselam, Raymond-Jonas Ngendabanka, Pia K. Muchaal, Kanchana Amaratunga, Aanchal Mishra, Rashmi Narkar, Anna-Louise Crago, Tanya Lary

**Affiliations:** 1 Antimicrobial Resistant Task Force, Public Health Agency of Canada, Ottawa, Ontario, Canada; 2 Applied Public Health Sciences Directorate, Public Health Agency of Canada, Ottawa, Ontario Canada; Fayetteville State University, UNITED STATES OF AMERICA

## Abstract

**Background:**

Antimicrobial resistance (AMR) is a growing global health threat that undermines the effectiveness of treatments and the sustainability of health systems. In 2015, Canada published its first AMR pathogen prioritization list, which laid the foundation for the Canadian Antimicrobial Resistance Surveillance System (CARSS). Since then, evolving resistance patterns, newly emerging pathogens, and enhanced surveillance capacity have prompted a comprehensive update to identify the most pressing AMR threats in the Canadian context.

**Objective:**

To undertake a systematic and reproducible prioritization process, leveraging nationally representative Canadian data, and to identify the most pressing AMR threats. This risk prioritization aims to inform surveillance strategies, which then lends itself to infection prevention and control measures, stewardship initiatives, and research and innovation directions.

**Methods:**

A total of 155 pathogens identified as potential threats to Canadians were assessed to determine whether AMR posed a significant concern. Pathogens selected for further evaluation underwent a multi-criteria decision analysis (MCDA) using Canadian data from 2017 to 2022. Nine prioritization criteria, including health equity introduced for the first time, were used to evaluate and rank pathogens based on their risk to public health. Weights were assigned to each criterion, informed by expert consensus, to reflect their relative importance. A sensitivity analysis was conducted to test the robustness of the rankings under different weighting scenarios, ensuring the reliability of the top-ranked AMR pathogens in Canada.

**Results:**

Twenty-nine AMR pathogens were ascertained as significant risks to Canadians and categorized into four tiers based on incidence, treatability, transmission, and health equity. Tier 1 pathogens, including Carbapenem-resistant *Enterobacterales, Candida auris*, Drug-resistant *Neisseria gonorrhoeae*, and Drug-resistant *Shigella spp*., pose the highest risk due to limited treatment options, potentially higher morbidity and mortality, and disproportionate impacts on marginalized populations. The prioritization of *N. gonorrhea* and Drug-resistant *Shigella spp.* along with the inclusion of *Mycoplasma genitalium* in Tier 2 highlight growing concerns in sexually transmitted infections (STIs). These findings underscore the need for continued enhanced surveillance, targeted interventions, and a health equity lens in AMR prioritization.

**Conclusion:**

This updated prioritization provides a robust, equity-informed framework to guide AMR surveillance and responses in Canada. It identifies high-impact pathogens and surveillance areas, offering a strategic tool to support public health action and research.

## Introduction

Antimicrobial resistance (AMR) is a critical global health challenge, threatening the effectiveness of treatments for bacterial, fungal, viral and parasitic infections and jeopardizing decades of medical progress [1]. The World Health Organization (WHO) has identified AMR as one of the top 10 global health threats [2]. This designation underscores the urgent need for coordinated, evidence-based strategies to mitigate its impact. In Canada, the 2019 *Council of Canadian Academies* (CCA) report [3], *When Antibiotics Fail: The Expert Panel on the Potential Socio-Economic Impacts of Antimicrobial Resistance in Canada*, estimated that AMR was responsible for approximately 14,000 deaths and imposed a $1.4 billion burden on the Canadian healthcare system in 2018 alone. The report projected that, if left unaddressed, AMR could lead to significant increase deaths annually by 2050, resulting in up to $120 billion in cumulative healthcare costs in Canada. Rising resistance patterns, the emergence of new AMR pathogens, and growing disparities in AMR-related health outcomes further reinforce the need for an adaptive, data-driven response.

A foundational step in addressing this need was the release of Canada’s first AMR Priority Pathogen Threats assessment in 2015 [4], which helped guide early surveillance and policy development. This work contributed to the creation of the Canadian Antimicrobial Resistance Surveillance System (CARSS), a national platform designed to synthesize AMR data from across the country [5–7]. Over time, CARSS has expanded its reach, now collating data from more than 13 AMR surveillance programs within the Public Health Agency of Canada (PHAC), each responsible for different pathogen types, sectors, or data sources [8–10]. This integration enables a more comprehensive understanding of the national AMR landscape. However, since the initial assessment, the AMR environment has evolved considerably, with emerging threats such as *Candida auris* [11,12] and *Mycoplasma genitalium* [13], as well as rising resistance in known pathogens, reinforcing the need to update and refine pathogen prioritization efforts. such as *Candida auris* (6) and *Mycoplasma genitalium* (7), alongside increasing resistance in some established pathogens, highlighting the need for a revised prioritization [10].

The 2025 AMR Priority Pathogen list builds upon the 2015 framework [4], aligning with international and domestic initiatives, including the WHO Priority Pathogens List [14,15] and the Pan-Canadian Action Plan on AMR [16]. Notably, Canada is the first country globally to incorporate health equity as a criterion in its AMR prioritization framework, recognizing the disproportionate burden of AMR on marginalized populations and ensuring that public health strategies address these disparities [16]. This novel approach reinforces Canada’s leadership in advancing an equitable public health response to AMR.

Advancements in surveillance capacity, including the implementation and recent expansion of AMRNet, a national laboratory-based surveillance system [9], have further strengthened the ability to monitor resistance trends. By integrating updated prioritization criteria, Canadian surveillance and epidemiological data, equity considerations, and rigorous validation methods, this revised prioritization serves as a tool to refine surveillance priorities, inform public health interventions (e.g., infection prevention and control, antimicrobial stewardship), and support innovation in research and development of therapeutics, diagnostics, and vaccines.

The purpose of this study is to provide a detailed overview of the methodology and results of the 2025 AMR prioritization exercise. To maintain a concise focus on the technical approach, interpretation of findings and its implications will be further explored in a forthcoming companion paper.

## Methods

The 2025 antimicrobial resistance (AMR) prioritization exercise builds upon the framework developed by the Public Health Agency of Canada (PHAC) in 2015 [4]. This iteration refines criteria and scoring values to reflect the evolving AMR landscape in Canada, incorporating advancements in data availability and shifts in public health priorities [5–7]. Multiple PHAC programs, including the Canadian Antimicrobial Resistance Surveillance System (CARSS), contributed to the design, validation, and implementation of this process [5–7].

### Development of expert working and advisory groups

PHAC convened a small advisory group, referred to throughout this paper as the PHAC AMR Internal Advisory Group (n = 10), comprised of AMR experts within the agency to provide strategic guidance throughout the prioritization process. This advisory group included epidemiologists from various AMR programs, laboratory microbiologists, modeling experts, and clinicians, ensuring a well-rounded, multidisciplinary approach to the prioritization exercise.

A separate 2025 AMR Priority Pathogen Working Group was also established, comprising members from CARSS (n > 20), which includes representation of the 13 surveillance programs across the agency working on AMR, as well Health Canada’s Pathogens of Interest team (n = 4), and other PHAC programs with expertise in prioritization and methods (n = 5). The collective input of both the AMR internal advisory group and the AMR Priority Pathogen Working Group contributed expertise in selecting the pathogens, refining prioritization criteria, scoring, weighting, and validating results, ensuring alignment with Canadian public health priorities and the integration of multidisciplinary perspectives.

### Pathogen selection

Previous prioritization work, which included a comprehensive literature search by PHAC in 2024, identified 155 infectious disease risks in Canada. From this list, the AMR Priority Pathogen Working Group identified 68 pathogens (44%) that exhibited evidence of antimicrobial resistance (AMR). This determination was based on a combination of available Canadian surveillance data, a literature review (including peer-reviewed and grey literature), and international case reports. In several cases, pathogens were flagged due to laboratory-confirmed resistance mechanisms or documented clinical resistance globally.

To determine which of the 68 AMR pathogens would proceed to scoring and ranking, the AMR Priority Pathogen Working Group applied a set of screening criteria to ensure alignment with the Canadian context. These criteria assessed whether each pathogen could be adequately evaluated using available data from 2017 to 2022 and whether it could be scored across all prioritization criteria in the multi-criteria decision analysis (MCDA). Pathogens that lacked sufficient data to be meaningfully assessed, where evidence was too limited to populate the scoring framework, were excluded, as they would receive a default score of zero. Sufficient data was defined as having enough information to allow for meaningful quantification across the nine prioritization criteria used in the MCDA framework.

In cases where AMR had not yet been identified in Canada but was recognized internationally, particularly in regions frequently visited by Canadian residents, pathogens were still considered if they posed a plausible future risk. Plausibility was assessed based on geographic proximity (e.g., the United States, Caribbean) and travel frequency, using Statistics Canada data on the most commonly visited countries [17]. Where available, Canadian surveillance and outbreak reports were also reviewed to identify instances of AMR pathogens acquired through international travel or medical tourism. A foresight-informed screening was applied to determine whether such pathogens met the criteria for high-consequence or pandemic potential, as outlined by the World Health Organization [1,2,14], ensuring early-stage threats were not excluded prematurely.Pathogens were also excluded if they:

- Had no documented association with AMR in laboratory-confirmed human cases in Canada;
- Were considered extremely rare in Canada, defined as being reported at a rate of one or fewer human cases per 100,000 population or 1,000 patient-days every five years;
- Represented AMR infections, but no considered a human health concern.

In cases where AMR had not yet been identified in Canada but was recognized internationally, particularly in regions frequented by Canadians, these pathogens were still considered if they posed a plausible future risk. A foresight-informed screening was also applied to evaluate whether these pathogens met the criteria for high-consequence or pandemic potential, as outlined by the World Health Organization [14], to ensure early-stage threats were not excluded prematurely.

Finally, for inclusion in scoring and ranking, pathogens were required to demonstrate resistance to at least one empirically used or recommended treatment option in Canada. While *Clostridioides difficile* does not meet this definition, given that current treatment options remain effective, the PHAC AMR Internal Advisory Group recommended its inclusion due to its strong association with antimicrobial use and its continued relevance as an AMR-related public health indicator. As such, *C. difficile* was retained in the prioritization to reflect its importance within the Canadian surveillance context. Documentation to support these assessments was from peer-reviewed literature, open-access reports from public health organizations, internal PHAC datasets, provincial/territorial surveillance publications, and other sources such as government reports, global surveillance platforms (e.g., WHO, ECDC, and national laboratory networks) [3–11,13,14,16–26]. Pathogens that lacked sufficient evidence for scoring or were too rare to assess reliably were assigned to Tier 5 (not shown), indicating they were outside the scope of prioritization at this time.

### Criteria refinement

The nine prioritization criteria originally used in 2015 by Garner. et al. [4] were reviewed and compared to international AMR pathogen prioritization frameworks, including those from the World Health Organization (WHO) [14] and the Centers for Disease Control and Prevention (CDC) [27]. Adjustments were made to incorporate Canada-specific considerations, including the addition of a health equity criterion. These refinements were reviewed by the PHAC AMR expert internal advisory group and the Working Group to ensure they remained relevant to current public health priorities [16].

### Scoring and ranking

Each pathogen was assessed using nine prioritization criteria that reflect public health priorities in Canada (Table 1). The four-point scoring scale assigned values of 0 (negligible concern), 1 (low concern), 2 (intermediate concern), and 3 (high concern). The detection, preventability and health equity criteria, were scored using a three-point scale (Table 2).

**Table 1:** Canada’s AMR Pathogen Threats (2025): Pathogens by Priority Group Based on total Weighted Risk Scores out of 100 (n = 29).

Tier 1: High priority group (80–100^th^ percentile)	Tier 2: Medium-high priority group (60 to <80^th^ percentile)	Tier 3: Medium-low priority group (40 to <60^th^ percentile)	Tier 4: Low priority group (<40^th^ percentile)
Carbapenem-resistant Enterobacterales (97)	Drug-resistant *Shigella* spp. (70)	Clindamycin-resistant Invasive Group A *Streptococcus* (61)	Drug-resistant *Haemophilus influenzae* (58)
Drug-resistant *Neisseria gonorrhoeae* (92)	*Mycoplasma genitalium* (70)	Drug-resistant Influenza A (61)	Drug-resistant *Helicobacter pylori* (58)
Carbapenem-resistant *Pseudomonas aeruginosa* (84)	Drug-resistant *Streptococcus pneumoniae* (65)	Drug-resistant Human immunodeficiency virus (59)	Drug -resistant *Candida* spp., excluding *Candida auris* (57)
Carbapenem-resistant *Acinetobacter spp.* (83)	Methicillin-Resistant *Staphylococcus aureus* (63)	Drug-resistant Group B *Streptococcus* (59)	Drug-resistant *Campylobacter* spp. (56)
*Candida auris* (75)	Vancomycin-resistant *Enterococcus* spp. (62)	*Clostridioides difficile* (59)	Drug-resistant *Bacteroides* spp. (55)
Extended spectrum B-lactamase-producing Enterobacterales (71)	Drug-resistant *Salmonella* spp. (Non-typhoidal) (62)	Multi-drug resistant *Mycobacterium tuberculosis* (59)	*Ureaplasma* spp. (53)
		Drug-resistant *Aspergillus* spp. (59)	Drug-resistant *Treponema pallidum* (53)
		Drug-resistant *Salmonella* spp. (Typhoidal) (59)	Drug-resistant *Chlamydia trachomatis* (46)
			Drug-resistant Pulmonary non-tuberculosis *Mycobacteria* (45)

**Note**: Scores were rounded to the nearest whole number.

**Table 2:** Prioritization Criteria, Scoring Definitions, and Weighting for Canada’s 2025 AMR Priority Pathogen List.

Criteria	Definition	Scoring Values				Weight
		0		1	2	3
Incidence	Average number of resistant cases per year in Canada between 2017–2022	<10 cases	10-100 cases per year	101-500 cases per year	>500 cases per year	11
Trend	The change in proportion of resistant cases (%) in Canada from 2017–2022	No case **OR** Too few cases	Decreasing trend	Stable trend at a level >0	Increasing trend	10
Mode of Transmission	Mechanism of spread from organisms to susceptible human host resulting in infection	Spread between person is rare but can occur	Can spread readily in healthcare settings	Can spread readily in the community	Airborne transmission **OR** Indirect contact	11
Case Fatality Ratio (%)^α^	Case fatality currently associated with the resistant pathogen	<5%	5-29%	30-60%	>60%	13
Morbidity	The severity of the disease considering clinical course and long-term sequelae	Asymptomatic illness	Infection causes mild disease that may require a visit to the doctor’s office	Infection causes disease that are rarely life-threatening but can require inpatient care	Infection causes life threatening illness	14
Treatability	Availability and effectiveness of recommended first or alternative treatment options in Canada	Majority of infections resolve on their own and does not require treatment^*^	Recommended first-line treatment options remains effective^**^	Resistant^***^ to at least one recommended first-line treatment options	Decreased effectiveness^****^ and/or access to recommended first-line and alternate treatment options	14
Detection	Capacity to detect occurrence of the disease and/or pathogen in an accurate and timely matter in Canada	–	Captured within an existing enhanced national surveillance system	Captured within an existing passive or limited national surveillance system or at a P/T level (P/T surveillance-research level)	Not captured by a formal surveillance system	11
Equity: Exposure to Disease	Does this disease disproportionately affect key populations^β^ that face social or economic marginalization or structural barriers to health	–	No, the disease does not disproportionally affect any of the key populations for health equity	Yes, the disease disproportionally affects at least one of the key populations for health equity	Yes, the disease disproportionally affects all or majority of the key population for health equity	9
Preventability	Ability to prevent the disease in humans, in Canada, considering public health measures and actions	–	Disease is vaccine preventable	Disease is not vaccine preventable **BUT** Disease can be prevented through effective well established and available measures **AND** Behavioral changes requires compliance by general public and public health professionals	Disease is not vaccine preventable **AND** Disease cannot be prevented through effective well established and available measures	9

^**α**^CFR % were derived using a combination of sources, including national surveillance data, public health information sheets, and peer-reviewed studies.

These thresholds for Treatability were defined using a combination of evidence-based data, including Canadian surveillance findings and peer-reviewed literature as well as expert judgment provided by the PHAC AMR SME group:^:^

*Nearly all infections resolve spontaneously without treatment.

**Nearly all infections are susceptible recommended first-line treatment options.

***Approximately ≥10% of isolates (of specific pathogen) are resistant to at least one recommended first-line treatment.

*****Approximately ≥10% of isolates (of specific pathogen) are resistant to both at least one first-line treatment and one alternative treatment option. This score also includes cases where resistance to the first-line treatments exists, in addition to barriers to access exists to alternative or more reserved treatment options, further complicating patient management and care.

βKey Populations include the following: Indigenous communities, people who inject drugs (PWID) or use drugs, sex workers, gbMSM (gay, bisexual, and other men who have sex with men), unhoused individuals, new immigrants and refugees from conflict-affected or disaster-affected regions.

**Note**: Weights were rounded to the nearest whole number.

Scoring was based on available Canadian surveillance data from 2017 to 2022, including both publicly available and internal sources. When Canadian data were limited or unavailable, peer-reviewed publications, grey literature, and other credible sources, such as open-access public health databases, and international agency reports (e.g., WHO, CDC) [14,27] were consulted. In such cases, expert opinion from PHAC AMR internal advisory across PHAC program areas was used to complement the evidence base. All scores were reviewed and validated to ensure consistency and accuracy across pathogens.

### Data sources and quality assessment

Whenever possible, national-level PHAC surveillance data were used to ensure comprehensive coverage [5–10,21,23,28–30]. In cases where national data were unavailable, provincial and territorial (P/T) datasets were incorporated [12,29–34]. The health equity criterion was assessed using a combination of Canadian surveillance data and peer-reviewed studies to identify key populations who are disproportionately affected by AMR pathogens and who face social or economic marginalization or structural barriers to health, including Indigenous communities, people who inject drugs (PWID) or use drugs, sex workers, gay, bisexual, and other men who have sex with men (gbMSM), unhoused individuals, new immigrants and refugees from conflict-affected or disaster-affected regions [3,8,21,24–26,31,34–36].

Treatability assessments were conducted using available Canadian clinical guidelines, prioritizing national and provincial/territorial (P/T) recommendations while limiting reliance on international sources [37–39]. However, it is important to note that many Canadian guidelines incorporate recommendations directly from the Infectious Diseases Society of America (IDSA) [40] and the U.S. Centers for Disease Control and Prevention (CDC) [27].

Most pathogens were evaluated using AMR pathogen-specific Canadian guidelines [3,6,10,13,16,18–20,22,31,34,35,37]. Where such guidance was unavailable, either a pathogen-specific or syndromic treatment guidelines was referred to [11–13,19,20,38,41–46]. For instance, syndromic guidelines were used to assess pathogens: *Aspergillus spp*. [44], *Haemophilus influenzae* type B [34], *Helicobacter pylori* [31,45], *Bacteroides spp*. [28], non-tuberculous *Mycobacteria* (NTM) [37], and enteric pathogens (excluding drug-resistant *Shigella spp*.). Whereas, *Ureaplasma spp*., HIV, *Chlamydia trachomatis*, and *Treponema pallidum* were assessed using pathogen-specific guidelines available through Canadian public health sources [19,20,46].

To ensure consistency, transparency, and clinical relevance, scoring thresholds for treatment efficacy were defined by the PHAC AMR Internal advisory group. These thresholds were based on a combination of current Canadian surveillance data (2017–2022), peer-reviewed studies, and expert consensus to ensure that assessments reflected both real-world treatment outcomes and emerging resistance concerns.

This combined approach allowed the prioritization team to identify which score was appropriate for each AMR pathogen, using the best available Canadian evidence and clinical judgment. The following categories were applied:

- **Score 0** – Nearly all infections (by specific pathogen) resolve spontaneously without treatment.
- **Score 1** – Nearly all infections (by specific pathogen) are susceptible to the recommended first-line treatments.
- **Score 2** – Approximately 10% or more isolates (of specific pathogen) are resistant to at least one recommended first-line treatments.
- **Score 3** – Approximately 10% or more isolates (of specific pathogen) are resistant to both a recommended first-line treatments and an alternative treatment option. This score also includes cases where resistance to the first-line treatments exists, in addition to barriers to access to alternative or more reserved treatment options, further complicating patient management and care.

Given the absence of a standardized resistance threshold for specific pathogen-drug combinations in Canada, this study adopted a conservative 10% resistance threshold, a stricter approach compared to the WHO’s ≥20% threshold. This lower threshold was selected to support early detection of emerging resistance trends, ensuring timely public health responses.

To ensure consistency in decision-making, data sources were categorized into three quality levels based on their scope, reliability, and comprehensiveness. Reliability was evaluated by assessing the credibility of the data source, the recency of data (i.e., 2017–2022), frequency of data collection (e.g., continuous vs. ad hoc), the presence of standardized reporting procedures, and the availability of laboratory-confirmed resistance data. Sources with validated methodologies, consistent reporting across multiple jurisdictions, and peer-reviewed or programmatically vetted outputs were considered more reliable.

- **Very Good**: Data used to evaluate the pathogen against the 9 criteria were derived mainly from Canadian sources, primarily from national surveillance programs, where the AMR pathogen was well-characterized in the Canadian context with high certainty in scoring.
- **Good**: Data used to evaluate the pathogen against the 9 criteria were derived mainly from Canadian sources, but primarily obtained from provincial or municipal surveillance programs or from data reported by the majority of provinces and territories (P/Ts). These AMR pathogens were not monitored by an official national surveillance system or dedicated resources for systematic monitoring.
- **Fair:** Data used to evaluate the pathogen against the 9 criteria were derived mainly from non-Canadian sources, where the disease etiology was well understood but with moderate confidence in estimates. This category also included historical or limited-quality Canadian data, where uncertainties in scoring existed due to insufficient robust evidence. In the absence of systematic monitoring or comprehensive domestic data, scoring was further supported by expert opinion from PHAC AMR internal advisory group and pathogen-specific subject matter experts across PHAC program areas. These insights were used to contextualize international findings within the Canadian public health landscape, helping to inform scoring decisions while acknowledging a lower level of empirical certainty decisions while acknowledging a lower level of empirical certainty.

### Weighting approach and sensitivity validation

A rigorous sensitivity analysis was conducted to evaluate the robustness of the prioritization results and account for potential data uncertainties. This analysis aimed to validate the consistency of rankings under different methodological conditions and ensure that the prioritization framework was not disproportionately influenced by specific criteria or weighting choices. Three complementary approaches were applied to strengthen the reliability of the findings:

The first approach utilized a MCDA algorithm, where the primary weighted sum model, used for ranking pathogens, was validated against an alternative outranking method. The outranking method, which is less compensatory, assessed pathogens based on their relative performance across multiple criteria rather than aggregating scores. In a compensatory model, high performance in one criterion can offset poor performance in another; however, the outranking method does not allow this kind of compensation. Instead, it focuses on whether one pathogen consistently dominates another across the criteria. This ensured that rankings were not unduly driven by a single high-scoring criterion, reinforcing the stability of high-priority classifications.

The second approach employed rank stability analysis, applying a pairwise comparison algorithm to examine the impact of modifications to individual criterion weights on pathogen rankings. This analysis identified the degree to which changes in weighting would alter the prioritization of specific pathogens, particularly those in Tier 1, allowing for an assessment of ranking resilience. This approach provided insights into the sensitivity of rankings to shifts in public health priorities and emerging AMR threats.

The third approach incorporated an expert-informed weighting model, in which the members of the PHAC AMR internal advisory group independently assigned weights to the nine prioritization criteria based on their expertise and experience. This alternative weighting scheme was used to assess how rankings might shift under different decision-making perspectives. By integrating professional judgment, this method further validated the robustness of the prioritization framework and helped ensure alignment with real-world public health priorities.

Together, these validation methods confirmed that the prioritization framework remained stable across multiple weighting models and methodological approaches, ensuring that high-risk AMR pathogens were consistently ranked as top priorities.

### Scoring and tiering

The total risk score for each pathogen was calculated by multiplying individual criterion scores by their assigned weights and summing the results. Based on percentile groupings, AMR pathogens were classified into four priority tiers:

- **Tier 1** (80–100th percentile): Highest priority
- **Tier 2** (60–80th percentile): Medium-high priority
- **Tier 3** (40–60th percentile): Medium-low priority
- **Tier 4** (<40th percentile): Low priority

Additionally, a Tier 5 category was designated for AMR pathogens that did not proceed to the scoring and ranking phase, either due to exclusion during the screening stage or the inability to quantify them against the assessment criteria. While these pathogens were identified as having potential AMR concerns, they were not ranked in this analysis and are therefore not presented in the final results or discussed in this manuscript.

### Statistical analysis

Data collection and repository management were conducted using Microsoft Office Excel 2023, while scoring aggregation, statistical analyses, and sensitivity testing were performed using R Core Team [47]. Descriptive analyses were conducted to contextualize the prioritization findings.

The proportion of AMR pathogens resistant to at least one first-line treatment option was determined by identifying all pathogens that received a score of 2 or 3 on the Treatability criterion (Table 2). The total number of these pathogens was then divided by the overall number of pathogens assessed to quantify the burden of resistance to first line therapy.

The proportion of pathogens disproportionately impacting marginalized populations was calculated by identifying pathogens that scored 2 or 3 on the Equity Exposure criterion (Table 2), using Canadian surveillance data and peer-reviewed studies. The total number of these pathogens was then divided by the overall number of AMR pathogens (n = 29) on the priority list to assess the extent to which health equity considerations factored into the prioritization exercise.

## Results

**Fig 1:**
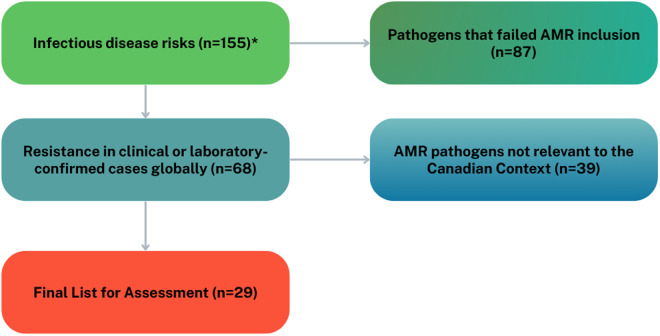
Flow Chart of the AMR Pathogen Selection Process. * The initial identification of infectious disease risks was conducted by a separate team within the PHAC, as part of a broader risk prioritization work examining all infectious diseases, not just AMR threats.

The 2025 AMR prioritization exercise initially identified 68 pathogens with demonstrated AMR based on global clinical and laboratory-confirmed data [2,14,15]. Following a series of inclusion screenings, 29 of these pathogens were selected for full evaluation using a comprehensive, data-driven methodology (Fig 1; Table 1) [4–6]. Selection criteria prioritized pathogens with evidence of resistance in Canada, availability of national surveillance data, and feasibility of assessment across the full set of prioritization criteria (Table 2). Pathogens that did not meet these conditions were excluded from the scoring and ranking process and assigned to Tier 5 (not shown), indicating insufficient evidence for national prioritization at this time.

The 2025 iteration built on the 2015 methodology, which screened 32 AMR pathogens, and reflects advancements in data availability, and surveillance infrastructure in Canada [4–6]. In 2015, only 11 pathogens were assessed using Canadian data; the remainder relied heavily on international sources and expert opinion due to major gaps in national AMR surveillance (3). In contrast, the current prioritization benefited from a broader foundation of domestic data and surveillance systems, including both national and provincial/territorial sources [5–10,21,23,28–30].

Of the 29 AMR pathogens assessed, 26 were supported by data classified as Very Good or Good, indicating a high degree of confidence in their characterization based on Canadian data (Table 3) [5,6,8–10]. These improvements are largely attributable to the expansion of AMR surveillance across Canada, supported by programs such as AMRNet [9], the Canadian Nosocomial Infection Surveillance Program (CNISP) [10], and the Enhanced Surveillance of Antimicrobial-Resistant Gonorrhea (ESAG) [8]. Additional advancements in laboratory capacity, epidemiological and disaggregated data collection, and reporting mechanisms have collectively strengthened Canada’s ability to characterize AMR threats.

**Table 3:** Data Sources and Quality Assessment for Canada’s 2025 AMR Priority Pathogen Threats (n = 29).

Quality of Evidence	% (proportion)	Data Sources*
Very Good	45% (13/29)	ARNI, AMRNet, CBTLSS, CIPARS, CNISP, ESAG, eStrep, and FoodNet Canada, GASP, PHAC STI guidelines, PHAC Pathogen safety Sheets, Peer-reviewed studies, P/T surveillance, MUMS
Good	48% (14/29)	May include data sources from “Very Good” but heavily relied on P/T individual surveillance systems, and Canadian peer-reviewed target studies
Fair	7% (2/29)	Includes limited Canadian and P/T data and populated with International surveillance systems/studies with comparable health infrastructure to Canada

*MUMS = Anti-infective Guidelines for Community-acquired Infections; Full program name: ARNI = Antimicrobial Resistance and Nosocomial Infections; AMRNet = Antimicrobial Resistance Network; CBTLSS = Canadian Tuberculosis Laboratory Surveillance System; CIPARS = Canadian Integrated Program for Antimicrobial Resistance Surveillance; CNISP = Canadian Nosocomial Infection Surveillance Program; ESAG = Enhanced Surveillance of Antimicrobial-Resistant Gonorrhea; eStrep = National Laboratory Surveillance of Invasive Streptococcal Disease in Canada, GASP = Gonococcal Antimicrobial Surveillance Program.

Pathogens were categorized into one of four priority tiers based on their overall weighted scores, moving away from absolute numerical rankings. This approach emphasizes the collective importance of high-impact AMR threats, particularly those in Tiers 1 and 2, where public health resources and interventions can have the greatest impact. The evaluation process incorporated nine prioritization criteria, each weighted according to its public health significance (Table 2). Fig 1 presents a breakdown of how each criterion contributed to the total weighted score, with Treatability, Transmission Potential, and Morbidity emerging as key drivers of tier placement.

The highest-priority AMR pathogens, classified as Tier 1, pose the greatest public health risk and represent the top 20% of rankings. These include carbapenem-resistant *Enterobacterales* (CRE), carbapenem-resistant *Pseudomonas aeruginosa* (CRPA), carbapenem-resistant *Acinetobacter spp.* (CRA) (25, 48), *Candida auris* [11,12,30,32,33], ESBL-producing *Enterobacterales* [23], and drug-resistant **Neisseria gonorrhoeae* [*8,18,22,25*]*. High rankings for CRE, CRPA, and CRA were primarily driven by treatability challenges, with limited or absent treatment options, including barriers to access, and the more severe disease outcomes associated with these infections.

*Candida auris*, which was not included in the 2015 AMR threat list [4], has now emerged as a critical public health threat due to its resistance to multiple anti-fungals, and its ability to rapidly develop resistance, multi-drug resistant (MDR) isolates, ease of and increase in transmission in healthcare settings, and high morbidity [11,12]. The first case in Canada was reported in 2012, with resistance first documented in 2017 [12].

Drug-resistant *Neisseria gonorrhoeae* was also classified as a Tier 1 pathogen, due to rising resistance to first-line therapies and its disproportionate burden among gbMSM and northern communities [8,18,22,25]. Similarly, drug-resistant *Shigella spp.* was categorized as Tier 2 and is increasingly identified as an STI, particularly among gbMSM [29]. Many cases are linked to clusters of multidrug-resistant (MDR) and extensively drug-resistant (XDR) strains [26,29,48].

**Fig 2:**
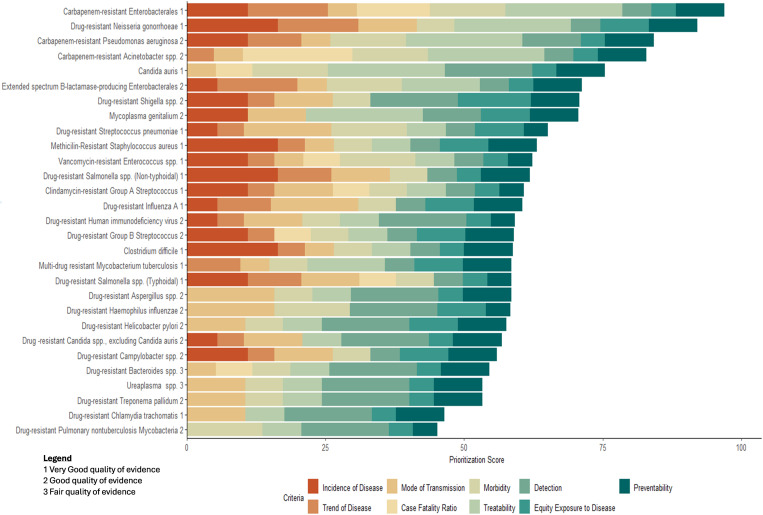
Contribution of Individual Weighted Criteria to the Total Score of Canada’s 2025 AMR Priority Pathogen Threats (n = 29).

*Mycoplasma genitalium* was also ranked as a Tier 2 pathogen, reflecting growing concerns over STI-related AMR, diagnostic limitations, and limited treatment options (Fig 2) [13].

A sensitivity analysis confirmed the robustness of the prioritization results. An alternative pairwise comparison algorithm demonstrated that rankings remained stable for key Tier 1 pathogens, including CRE, *C. auris*, and drug-resistant *N. gonorrhoeae*. Rank stability analysis showed that most Tier 1 pathogens were resilient to variations in individual criterion weights, though drug-resistant *N. gonorrhoeae* showed sensitivity to changes in the Equity Exposure weight, reinforcing its strong association with marginalized groups. Importantly, the final weighting used in the prioritization was strongly aligned with the expert-informed weighting model. Despite being developed independently, the two weighting approaches produced very similar distributions of weights across the nine criteria.

## Discussion

The 2025 prioritization exercise underscores critical AMR pathogen trends in Canada and reveals several potential implications for ongoing surveillance and public health policy, planning, and interventions. By applying a structured, data-driven framework grounded in Canadian surveillance data, the exercise highlights not only which pathogens pose the greatest threat to Canadians, but also how and where these threats are manifesting(i.e., community vs healthcare setting), offering a clear roadmap for action.

One of the key findings of this prioritization exercise is that 45% of the assessed AMR pathogens disproportionately impact populations experiencing social or economic marginalization and structural barriers to health [3,19,21,23,25,26,31,34,35]. These populations include Indigenous communities, PWID, sex workers, gbMSM, individuals experiencing homelessness, and newcomers such as refugees or immigrants from conflict- or disaster-affected regions [3,19,21,23,25,26,31,34,35]. By explicitly integrating health equity as a prioritization criterion for the first time, this exercise allowed for a more inclusive and contextually grounded assessment of AMR threats in Canada. Rather than relying solely on traditional metrics such as incidence or mortality, this approach recognizes that vulnerability to AMR is shaped by broader social determinants of health. Prioritizing equity in AMR planning not only strengthens the relevance of public health responses, but also ensures that interventions are better tailored to address disparities in exposure, access to care, and treatment outcomes, ultimately contributing to a more equitable and effective response to emerging AMR threats.

In terms of treatability of these pathogens, approximately 28% of the AMR pathogens were found to be resistant to at least one recommended first-line antimicrobial therapy currently used in Canada. This finding has direct public health implications, pointing to the need for ongoing review and updates to treatment guidelines, increased availability and access to alternative and reserve treatments, and sustained antimicrobial stewardship and IPC efforts. It also reinforces the importance of surveillance systems that monitor not only resistance rates but also treatment efficacy and outcomes across different population groups and regions.

The results also show that while 31% of prioritized pathogens are primarily associated with healthcare settings, approximately 70% are linked to community transmission (39, 46) [35,43]. This distribution challenges the perception of AMR as solely a hospital-based issue and calls for expanded prevention and control strategies that reach beyond traditional healthcare environments [3,19,21,23,25,26,31,34,35]. Notably, the rise in detection of CPOs and ESBL-producing *Enterobacterales* in community-acquired enteric infections highlights that these resistance threats are no longer confined to acute-care settings [23,30,32,33]. These findings reinforce the need for strengthened surveillance and IPC strategies that extend into primary care, long-term care, and community settings.

Newly prioritized pathogens such as *Candida auris* and *Mycoplasma genitalium* reflect the evolving nature of the AMR landscape in Canada. Their inclusion underscores critical challenges in diagnostic capacity, treatment availability, and surveillance coverage—areas that require ongoing investment and innovation [11–13]. Conversely, the downward classification of *C.difficile* and Methicillin-resistant *Staphylococcus aureus* (MRSA) illustrates how enhanced surveillance, infection prevention and control measures, and antimicrobial stewardship can help mitigate the burden of resistant infections over time [24,28,35,49].

While MRSA remains a globally recognized and important AMR threat, its shift from Tier 1 in 2015 to Tier 2 in 2025 reflects a change in assessment scope. In 2015, prioritization was based solely on bloodstream infections, whereas the 2025 assessment considered all MRSA infection types [35,49]. This broader approach provides a more comprehensive understanding of MRSA’s impact in Canada and better aligns with current surveillance capabilities and the clinical landscape.

It is important to note that *C. difficile* is not typically considered an AMR pathogen in Canada because, although it can exhibit resistance to many antibiotics, it remains susceptible to those most commonly recommended for treatment [24,28]. As such, current Canadian treatment regimens remain effective. Nevertheless, it was included in this prioritization exercise because *C. difficile* infections often arise in patients who have experienced antibiotic overuse or misuse, making it a useful indicator of broader AMR pressures [24,28]. While it is not classified as an AMR pathogen per se, its strong association with antimicrobial exposure highlights a critical pathway through which resistance emerges and spreads. As such, monitoring *C. difficile* remains an important consideration in Canada’s efforts to combat AMR.

Taken together, the findings emphasize the need for a multipronged public health response. Key areas of focus should include expanding AMR surveillance infrastructure, particularly in community settings, integrating equity-informed policies into AMR planning, and developing flexible, evidence-based interventions that evolve alongside resistance patterns. These actions will be essential to maintaining the effectiveness of existing antimicrobials and safeguarding public health in the face of rapidly evolving AMR threats.

A key limitation of this prioritization exercise is the variability in AMR surveillance systems and data collection protocols across different pathogens within different programs. Surveillance approaches differ in scope, methodology, and consistency, even at the national level, where data collection is shaped by differing provincial and territorial (P/T) processes. Within P/T jurisdictions, regional differences in antimicrobial susceptibility testing (AST) methods, including variations in antibiogram protocols, further contribute to inconsistencies in data availability and quality. These differences can limit the ability to produce fully harmonized national estimates for certain pathogens.

Another limitation lies in the scope of the treatability assessment, which was based primarily on Canadian clinical guidelines, supplemented where necessary by guidelines from the Infectious Diseases Society of America (IDSA) and the Centers for Disease Control and Prevention (CDC) [18,24,27,38,40,41,45,46,49]. While this ensured alignment with current clinical practice, a more granular approach, accounting for syndromic presentations, infection site, resistance mechanism, and patient-specific characteristics, was not feasible within the framework of this analysis. WHO guidelines were also excluded, as they predominantly follow a syndromic model rather than a pathogen-specific approach, although they may still be referenced by Canadian clinicians [14,15]. As resistance patterns continue to evolve, future iterations of this exercise may benefit from incorporating more dynamic and flexible clinical inputs that reflect these complexities.

Importantly, this prioritization exercise relied on data from the 2017–2022 period. While this five-year range was selected to ensure consistency and data quality across sources, it also introduces a natural time lag. Given that AMR trends are dynamic and can evolve rapidly, recent developments, such as shifts in resistance prevalence, updates to treatment guidelines, or the emergence of new pathogens, may not be fully reflected in this assessment. To maintain relevance and support evidence-based decision-making, national prioritization exercises should be revisited every three to five years. Routine updates, supported by ongoing surveillance and stakeholder input, are essential to ensure that policy, research, and public health interventions remain aligned with the evolving AMR landscape in Canada.Additionally, all AMR pathogens in this exercise were assessed based on infected cases, with the exception of *Candida auris*, which included both infected and colonized cases. This approach reflects how *C. auris* is reported nationally and acknowledges its high transmissibility in healthcare settings, where colonization often precedes infection [11,12]. Including colonized cases better reflects the full scope of potential infections and transmission risk. Although *C. auris* has been under enhanced national surveillance since 2019, the majority of cases during the 2017–2022 study period were not captured through that system [11,12]. As such, it was not scored as 1 in the Detection criteria – captured with an existing enhanced surveillance system.

A final challenge is the limited availability of disaggregated health equity data in national surveillance systems. The impact of AMR on marginalized and disproportionately affected populations is often better characterized through targeted studies, which fall outside routine surveillance. Given that treatment and patient care remain the primary priorities in most P/T health systems, AMR surveillance – including the collection of equity-related data – is often secondary [3,19,21,23,25,26,31,34,35]. Strengthening Canada’s surveillance frameworks to include more systematic equity data collection will be critical for developing tailored and inclusive AMR responses in the future.

## Conclusion

The findings of this study highlight the evolving landscape of AMR threats in Canada and reinforce the importance of a systematic, evidence-informed approach to public health risk prioritization. Since the original 2015 assessment, Canada has made substantial progress in expanding national surveillance capacity, identifying emerging pathogens of concern, and integrating health equity into decision-making frameworks. These advances have enabled a more comprehensive and inclusive understanding of AMR risk across diverse populations and settings.

Despite these gains, notable challenges remain, particularly in addressing surveillance gaps, including access to more timely data and reporting, and capturing disaggregated equity-related information. The explicit inclusion of health equity as a prioritization criterion marks an important advancement, revealing the disproportionate burden of AMR on socially and economically marginalized populations and underscoring the need for targeted interventions to address these disparities.

This prioritization framework is not static. It is designed to adapt to an ever-changing AMR landscape and can serve as a foundational tool to inform national surveillance strategies, support IPC and antimicrobial stewardship activities, and guide investments in public health research and development. The framework can inform research and development priorities in the realm of novel therapeutics, vaccines and diagnostics, as well as potentially identify barriers to their access in order to help re-evaluate existing processes and policies.

While Tier 1 and Tier 2 pathogens require more attention at present, pathogens in Tiers 3 and 4 must not be overlooked. The dynamic nature of AMR means that lower-priority pathogens today may evolve into significant threats tomorrow, particularly as resistance patterns shift, diagnostics improve, and/or treatment and/or preventive options become limited or infective. Continued monitoring across all tiers is therefore essential to ensure early detection and timely intervention.

Together, these findings provide a forward-looking roadmap for strengthening Canada’s response to AMR through sustained surveillance infrastructure, intergovernmental collaboration, equity-informed planning, and flexible, data-driven public health action.

## Supporting information

S1 File Scoring Framework and References. (DOCX)

S2 File Quality of Evidence Evaluation of Sources. (DOCX)

## Abbreviations

AMR: Antimicrobial Resistance
AMRNet: Antimicrobial Resistance Network
APHS: Applied Public Health Sciences Directorate
ARNI: Antimicrobial Resistance and Nosocomial Infections
AST: Antimicrobial Susceptibility Testing
CARSS: Canadian Antimicrobial Resistance Surveillance System
CBTLSS: Canadian Bacterial and Tuberculosis Laboratory Surveillance System
CDC: Centers for Disease Control and Prevention
CIPARS: Canadian Integrated Program for Antimicrobial Resistance Surveillance
CNISP: Canadian Nosocomial Infection Surveillance Program
CPO: Carbapenem-Producing OrganismCRE, Carbapenem-resistant Enterobacterales
CRA: Carbapenem-resistant Acinetobacter spp.
CRPA: Carbapenem-resistant Pseudomonas aeruginosa
ESAG: Enhanced Surveillance of Antimicrobial-Resistant Gonorrhea
eStrep: National Labratory Surveillance of Invasive Streptococcal Disease in Canada
ESBL: Extended-Spectrum Beta-Lactamase-producing Enterobacterales
FoodNet Canada: Canadian Foodborne Disease Surveillance Network
GASP: Gonococcal Antimicrobial Surveillance Program
gbMSM: Gay, Bisexual, and other Men who have Sex with Men
MCDA: Multi-Criteria Decision Analysis
MRSA: Methicillin-resistant Staphylococcus aureus
NML: National Microbiology Laboratory
PHAC: Public Health Agency of Canada
P/T: Provincial and Territorial
PWID: People Who Inject Drugs
WHO: World Health Organization.

## References

[1] World Health Organization B. No time to wait: securing the future from drug-resistant infections. World Health Organization: Geneva, Switzerland. 2019.

[2] World Health Organization B. Ten threats to global health in 2019 who.int: WHO; 2019. Available from: https://www.who.int/news-room/spotlight/ten-threats-to-global-health-in-2019

[3] Finlay BB, Conly J, Coyte PC, Dillon J-AR, Douglas G, Goddard E, et al. When antibiotics fail: the expert panel on the potential socio-economic impacts of antimicrobial resistance in Canada. 2019.

[4] Garner MJ, Carson C, Lingohr EJ, Fazil A, Edge VL, Trumble Waddell J. An assessment of antimicrobial resistant disease threats in Canada. PLoS One. 2015;10(4):e0125155. doi: 10.1371/journal.pone.0125155 25905797 PMC4408042

[5] Canada PHAo. Canadian Antimicrobial Resistance Surveillance System (CARSS) Report 2022. Canada.ca: 2022 2022-11-28. Report No.

[6] Canada PHAo. Canadian Antimicrobial Resistance Surveillance System (CARSS). 2024.

[7] Canada PHAo. Canadian Antimicrobial Resistance Surveillance System Report 2016. 2016 2016-09-12. Report No.

[8] Canada PHAo. Dashboard on the Enhanced Surveillance of Antimicrobial-resistant Gonorrhea system (ESAG): 2018 to 2023 2025. Available from: https://infobase-dev.com/ESAG/index.html

[9] Rudnick W, Mukhi SN, Reid-Smith RJ, German GJ, Nichani A, Mulvey MR, et al. Overview of Canada’s Antimicrobial Resistance Network (AMRNet): A data-driven One Health approach to antimicrobial resistance surveillance. Can Commun Dis Rep. 2022;48(11–12):522–8. doi: 10.14745/ccdr.v48i1112a05 38173468 PMC10760988

[10] Canadian Nosocomial Infection Surveillance P. Healthcare-associated infections and antimicrobial resistance in Canadian acute care hospitals, 2017–2021. Canada Communicable Disease Report. 2023;49(5):235. 38425696 10.14745/ccdr.v49i05a09 PMC10903608

[11] Canada PHAo. Candida auris: Infectious substances pathogen safety data sheet 2024. Available from: https://www.canada.ca/en/public-health/services/laboratory-biosafety-biosecurity/pathogen-safety-data-sheets-risk-assessment/candida-auris.html

[12] Ontario PH. Candida auris. 2023.

[13] CanadaPHAo. Mycoplasma Genitalium guide: Key information and resources 2021. Available from: https://www.canada.ca/en/public-health/services/infectious-diseases/sexual-health-sexually-transmitted-infections/canadian-guidelines/mycoplasma-genitalium.html

[14] World Health O. WHO bacterial priority pathogens list, 2024: bacterial pathogens of public health importance, to guide research, development, and strategies to prevent and control antimicrobial resistance: World Health Organization; 2024.

[15] World Health O. WHO fungal priority pathogens list to guide research, development and public health action: World Health Organization; 2022.

[16] Canada PHAo. Tackling Antimicrobial Resistance and Antimicrobial Use: A Pan-Canadian Framework for Action. Canada.ca: 2017 2017-09-05. Report No. 10.14745/ccdr.v43i11a01 PMC5764732 29770049

[17] Canada S. Travel and Tourism Statistics 2025. Available from: https://www.statcan.gc.ca/en/subjects-start/travel_and_tourism

[18] (NAC-STBBI) NACoSTaB-BI. Interim guidance for the treatment of uncomplicated gonococcal infections. 2024.

[19] Canada PHAo. Canadian guidelines on sexually transmitted infections. 2008.

[20] Canada PHAo. Chlamydia and LGV guide: Key information and resources. 2025.

[21] Golden A, Griffith A, Demczuk W, Tyrrell G, Kus J, McGeer A, et al. Invasive group A streptococcal disease surveillance in Canada, 2020. CCDR. 2022;48(9):407–14. doi: 10.14745/ccdr.v48i09a05 38106647 PMC10723789

[22] Infections NACoSTaB-B. Gonorrhea guide: Key information and resources Canada.ca 2024. Available from: https://www.canada.ca/en/public-health/services/infectious-diseases/sexual-health-sexually-transmitted-infections/canadian-guidelines/gonorrhea.html

[23] Karlowsky JA, Walkty A, Golden AR, Baxter MR, Denisuik AJ, McCracken M. ESBL-positive Escherichia coli and Klebsiella pneumoniae isolates from across Canada: CANWARD surveillance study, 2007–18. Journal of Antimicrobial Chemotherapy. 2021;76(11):2815–24. 34378029 10.1093/jac/dkab269

[24] Loo VG, Davis I, Embil J, Evans GA, Hota S, Lee C, et al. Association of Medical Microbiology and Infectious Disease Canada treatment practice guidelines for Clostridium difficile infection. Journal of the Association of Medical Microbiology and Infectious Disease Canada. 2018;3(2):71–92. doi: 10.3138/jammi.2018.02.13

[25] Sawatzky P, Lefebvre B, Diggle M, Hoang L, Wong J, Patel S. Antimicrobial susceptibilities of Neisseria gonorrhoeae in Canada, 2021. Canada Communicable Disease Report. 2023;49(9):388. 38463902 10.14745/ccdr.v49i09a05 PMC10919915

[26] Stefanovic A, Matic N, Ritchie G, Lowe CF, Leung V, Hull M, et al. Multidrug-Resistant Shigella sonnei Bacteremia among Persons Experiencing Homelessness, Vancouver, British Columbia, Canada. Emerg Infect Dis. 2023;29(8):1668–71. doi: 10.3201/eid2908.230323 37486309 PMC10370870

[27] CDC. Antibiotic resistance threats in the United States. US Department of Health and Human Services: Washington, DC, USA. 2019;1:67–100.

[28] Kajihara T, Yahara K, Kitamura N, Hirabayashi A, Hosaka Y, Sugai M. Distribution, trends, and antimicrobial susceptibility of Bacteroides, Clostridium, Fusobacterium, and Prevotella species causing bacteremia in Japan during 2011–2020: a retrospective observational study based on national surveillance data. 2023. 10.1093/ofid/ofad334 PMC10352651 37469615

[29] Ontario PH. Surveillance Report Shigella Antimicrobial Resistance. 2023.

[30] Services AH. Carbapenemaseproducing Organisms. (CPO) Protocol. 2025.

[31] Eng NF, Ybazeta G, Chapman K, Fraleigh NL, Letto R, Altman E, et al. Antimicrobial susceptibility of Canadian isolates of Helicobacter pylori in Northeastern Ontario. Can J Infect Dis Med Microbiol. 2015;26(3):137–44. doi: 10.1155/2015/853287 26236355 PMC4507839

[32] Ontario PH. Frequently Asked Questions Carbapenemase-Producing Enterobacteriaceae (CPE). 2019.

[33] Ontario) OAfHPaPPH. Provincial Infectious Diseases Advisory Committee. Evidence review and revised recommendations for the control of vancomycin-resistant enterococci in all Ontario health care facilities. Toronto, ON: 2019.

[34] Ulanova M, Tsang RSW, Nix EB, Kelly L, Shuel M, Lance B, et al. Epidemiology of invasive Haemophilus influenzae disease in northwestern Ontario: comparison of invasive and noninvasive H. influenzae clinical isolates. Can J Microbiol. 2023;69(6):219–27. doi: 10.1139/cjm-2022-0208 36753721

[35] Johnston B, Conly J. Community-associated methicillin-resistant Staphylococcus aureus: Continuing to evolve. Can J Infect Dis Med Microbiol. 2008;19(2):161–3. doi: 10.1155/2008/162493 19352446 PMC2605856

[36] Moore DL, Allen UD, Mailman T. Invasive group A streptococcal disease: Management and chemoprophylaxis. Paediatr Child Health. 2019;24(2):128–9. doi: 10.1093/pch/pxz039 30996606 PMC6462117

[37] Brode SK, Dwilow R, Kunimoto D, Menzies D, Khan FA. Chapter 8: Drug-resistant tuberculosis. Canadian Journal of Respiratory, Critical Care, and Sleep Medicine. 2022;6(sup1):109–28. doi: 10.1080/24745332.2022.2039499

[38] (SHSC) SHSC. Antimicrobial stewardship treatment guidelines 2024. Available from: https://sunnybrook.ca/content/?page=antimicrobial-stewardship

[39] Evans L, Rhodes A, Alhazzani W, Antonelli M, Coopersmith CM, French C, et al. Surviving Sepsis Campaign: International Guidelines for Management of Sepsis and Septic Shock 2021. Crit Care Med. 2021;49(11):e1063–143. doi: 10.1097/CCM.0000000000005337 34605781

[40] Tamma PD, Heil EL, Justo JA, Mathers AJ, Satlin MJ, Bonomo RA. Infectious Diseases Society of America 2024 Guidance on the Treatment of Antimicrobial-Resistant Gram-Negative Infections. Clin Infect Dis. 2024;:ciae403. doi: 10.1093/cid/ciae403 39108079

[41] File TM Jr. Clinical implications and treatment of multiresistant Streptococcus pneumoniae pneumonia. Clin Microbiol Infect. 2006;12 Suppl 3:31–41. doi: 10.1111/j.1469-0691.2006.01395.x 16669927

[42] Shankar-Hari M, Phillips GS, Levy ML, Seymour CW, Liu VX, Deutschman CS, et al. Developing a New Definition and Assessing New Clinical Criteria for Septic Shock: For the Third International Consensus Definitions for Sepsis and Septic Shock (Sepsis-3). JAMA. 2016;315(8):775–87. doi: 10.1001/jama.2016.0289 26903336 PMC4910392

[43] Health M. Anti-infective Guidelines for Community-acquired Infection: MUMS Health Clearinghouse; 2024.

[44] Patterson TF, Thompson GR 3rd, Denning DW, Fishman JA, Hadley S, Herbrecht R, et al. Practice Guidelines for the Diagnosis and Management of Aspergillosis: 2016 Update by the Infectious Diseases Society of America. Clin Infect Dis. 2016;63(4):e1–60. doi: 10.1093/cid/ciw326 27365388 PMC4967602

[45] Zanten SVv. TCMP Article: Management of Helicobacter pylori in 2023: who should be tested, treated, and how: UBC Continuing Professional Development; 2023. Available from: https://ubccpd.ca/tcmp-article-management-helicobacter-pylori-2023-who-should-be-tested-treated-and-how

[46] CATIE. The emergence of dual drug therapy 2024. Available from: https://www.catie.ca/treatmentupdate-229/the-emergence-of-dual-drug-therapy

[47] Team RDC. R: A language and environment for statistical computing. (No Title). 2010.

[48] Charles H, McCall H, Mason A, Foster K, Ma R, Jenkins C, et al. Spotlight on drug-resistant Shigella: raising awareness within general practice. Br J Gen Pract. 2023;73(729):187–8. doi: 10.3399/bjgp23X732537 36997218 PMC10049588

[49] Liu C, Bayer A, Cosgrove SE, Daum RS, Fridkin SK, Gorwitz RJ, et al. Clinical practice guidelines by the infectious diseases society of america for the treatment of methicillin-resistant Staphylococcus aureus infections in adults and children. Clin Infect Dis. 2011;52(3):e18-55. doi: 10.1093/cid/ciq146 21208910

