# Supplementary material for: Canada’s 2025 AMR priority pathogens: Evidence-based ranking and public health implications
Source: PLoS One. 2025 Sep 17;20(9):e0330128. doi: 10.1371/journal.pone.0330128 (PMC12443280; doi:10.1371/journal.pone.0330128)
Supplement: S1 File — (DOCX) [file pone.0330128.s001.docx]

**Scoring Framework and References: Data Sources Used to Assess Pathogen Across Nine Criteria**

**Criteria Legend**

1. **Incidence**
2. **Trend**
3. **Mode of Transmission**
4. **Case Fatality Ratio (%)**
5. **Morbidity**
6. **Treatability**
7. **Detection**
8. **Equity - Exposure to Disease**
9. **Preventability**

***To request additional information or to request access to data from internal resources, please find the contact information for the appropriate resources below:**

ARNI., 2023 (Internal) - [nml.arni-rain.lnm@phac-aspc.gc.ca](mailto:nml.arni-rain.lnm@phac-aspc.gc.ca)

CIPARS-CFEZID (Internal) - [cipars-picra@phac-aspc.gc.ca](mailto:cipars-picra@phac-aspc.gc.ca)

NMLB (Internal) – [nml@phac-aspc.gc.ca](mailto:nml@phac-aspc.gc.ca)

Irene Martin - [irene.martin@phac-aspc.gc.ca](mailto:irene.martin@phac-aspc.gc.ca)

ESAG - [sti-hep-its@phac-aspc.gc.ca](mailto:sti-hep-its@phac-aspc.gc.ca)

GASP - [nml.strepsti-lnm.strepits@phac-aspc.gc.ca](https://health-infobase.canada.ca/gasp/nml.strepsti-lnm.strepits@phac-aspc.gc.ca)

(Reference table has been split into two, to ensure readability one table includes criteria 1-5 and the other 6-9)

| AMR Pathogen | Criteria | | | | |
| --- | --- | --- | --- | --- | --- |
|  | 1 | 2 | 3 | 4 | 5 |
| *Candida auris* | ARNI, 2023, internal document – reader must request directly from program area | ARNI, 2023, internal document – reader must request directly from program area | PHAC., 2024., ([Candida auris Infection Prevention and Control in Canadian Healthcare Settings - Canada.ca](https://www.canada.ca/en/services/health/publications/diseases-conditions/candida-auris-infection-prevention-control-canadian-healthcare-settings.html)) | PHO., 2023 ([Candida auris](https://www.publichealthontario.ca/-/media/Documents/C/2023/candida-auris.pdf?rev=b5e9d4ef8a674326b737b34bb1ca690b&sc_lang=en)) | PHAC., 2024., ([Candida auris Infection Prevention and Control in Canadian Healthcare Settings - Canada.ca](https://www.canada.ca/en/services/health/publications/diseases-conditions/candida-auris-infection-prevention-control-canadian-healthcare-settings.html)) |
| Carbapenem-resistant Enterobacterales | CNISP, p41., 2023 ([ccdrv49i05a09-eng.pdf](https://www.canada.ca/content/dam/phac-aspc/documents/services/reports-publications/canada-communicable-disease-report-ccdr/monthly-issue/2023-49/issue-5-may-2023/ccdrv49i05a09-eng.pdf)) | CNISP - CARSS., 2024 ([Antimicrobial resistance: Seasonal update — Canada.ca](https://health-infobase.canada.ca/carss/amr/)) | CNISP, p.240., 2023 ([ccdrv49i05a09-eng.pdf](https://www.canada.ca/content/dam/phac-aspc/documents/services/reports-publications/canada-communicable-disease-report-ccdr/monthly-issue/2023-49/issue-5-may-2023/ccdrv49i05a09-eng.pdf)) | PHO., 2019 ([Frequently Asked Questions: Carbapenemase-Producing Enterobacteriaceae (CPE)](https://www.publichealthontario.ca/-/media/Documents/F/2019/faq-cpe.pdf?rev=ba71848e5acb41ab91a1d5e03b6d46dd&sc_lang=en)) | CARSS., 2022 ([Canadian Antimicrobial Resistance Surveillance System (CARSS) Report 2022 - Canada.ca](https://www.canada.ca/en/public-health/services/publications/drugs-health-products/canadian-antimicrobial-resistance-surveillance-system-report-2022.html#a2.3)) |
| Drug-resistant *Neisseria gonorrhoeae* | P, Sawatzky., et al., 2023 ([2017-2020: p. 391; 2021: p.390](https://www.canada.ca/content/dam/phac-aspc/documents/services/reports-publications/canada-communicable-disease-report-ccdr/monthly-issue/2023-49/issue-9-september-2023/ccdrv49i09a05-eng.pdf)) | CNISP - CARSS., 2024 ([Antimicrobial resistance: Seasonal update — Canada.ca](https://health-infobase.canada.ca/carss/amr/)) | PHAC., 2024 ([Gonorrhea guide: Risk factors and clinical manifestations - Canada.ca](https://www.canada.ca/en/public-health/services/infectious-diseases/sexual-health-sexually-transmitted-infections/canadian-guidelines/gonorrhea/risk-factors-clinical-manifestation.html#a2)) | CNISP-CARSS., 2024 ([Antimicrobial resistance: Seasonal update — Canada.ca](https://health-infobase.canada.ca/carss/amr/results.html?ind=12)) | PHAC., 2022., ([Gonorrhea guide: Risk factors and clinical manifestations - Canada.ca](https://www.canada.ca/en/public-health/services/infectious-diseases/sexual-health-sexually-transmitted-infections/canadian-guidelines/gonorrhea/risk-factors-clinical-manifestation.html#a3)) |
| Drug-resistant Invasive Group A Streptococcus | CNISP - CARSS., 2024 ([Antimicrobial resistance: Seasonal update — Canada.ca](https://health-infobase.canada.ca/carss/amr/results.html?ind=16)) | CNISP - CARSS., 2024 ([Antimicrobial resistance: Seasonal update — Canada.ca](https://health-infobase.canada.ca/carss/amr/)) | PHAC., 2024 ([Group A streptococcal diseases (Streptococcus pyogenes) - Canada.ca](https://www.canada.ca/en/public-health/services/diseases/group-a-streptococcal-diseases.html#a3)) | Andrew D.K. Nguyen., et al., 2023 ([The efficacy and safety of a shortened duration of antimicrobial therapy for group A Streptococcus bacteremia - ScienceDirect](https://www.sciencedirect.com/science/article/pii/S1201971222006506)) | CARSS., 2022 ([Canadian Antimicrobial Resistance Surveillance System (CARSS) Report 2022 - Canada.ca](https://www.canada.ca/en/public-health/services/publications/drugs-health-products/canadian-antimicrobial-resistance-surveillance-system-report-2022.html#a2.3)) |
| Drug-resistant Streptococcus pneumoniae | CARSS, 2022 ([Canadian Antimicrobial Resistance Surveillance System Report 2022](https://www.canada.ca/content/dam/phac-aspc/documents/services/publications/drugs-health-products/canadian-antimicrobial-resistance-surveillance-system-report-2022/canadian-antimicrobial-resistance-surveillance-system-report-2022.pdf)) | CARSS., 2024 ([Antimicrobial resistance: Seasonal update — Canada.ca](https://health-infobase.canada.ca/carss/amr/)) | PHAC., 2025, ([Invasive Pneumococcal Disease - Canada.ca](https://www.canada.ca/en/public-health/services/immunization/vaccine-preventable-diseases/invasive-pneumococcal-disease.html)) | Angel Vila-Corcoles., et al., 2009  ([1471-2334-9-36.fm](https://pmc.ncbi.nlm.nih.gov/articles/PMC2667188/pdf/1471-2334-9-36.pdf)) | PHAC., 2023., ([Invasive Pneumococcal Disease - Canada.ca](https://www.canada.ca/en/public-health/services/immunization/vaccine-preventable-diseases/invasive-pneumococcal-disease/symptoms.html)) |
| Drug-resistant *Aspergillus* spp. | **2000-2013:** Parent-Michaud, M., et al., 2019 ([OP-JANM190539 849..858](https://pmc.ncbi.nlm.nih.gov/articles/PMC7069474/pdf/dkz534.pdf))  **2018**: Cheng, M.P., et al., 2019 ([(PDF) Triazole Antifungal Susceptibility Patterns among Aspergillus Species in Québec, Canada](https://www.researchgate.net/publication/332030387_Triazole_Antifungal_Susceptibility_Patterns_among_Aspergillus_Species_in_Quebec_Canada))  **Hamilton**: Eta Ebasi Ashu., et al., 2017 ([Limited evidence of fungicide-driven triazole-resistant Aspergillus fumigatus in Hamilton, Canada](https://cdnsciencepub.com/doi/abs/10.1139/cjm-2017-0410)) | **2000-2013:** Parent-Michaud, M., et al., 2019 ([OP-JANM190539 849..858](https://pmc.ncbi.nlm.nih.gov/articles/PMC7069474/pdf/dkz534.pdf))  **2018**: Cheng, M.P., et al., 2019 ([(PDF) Triazole Antifungal Susceptibility Patterns among Aspergillus Species in Québec, Canada](https://www.researchgate.net/publication/332030387_Triazole_Antifungal_Susceptibility_Patterns_among_Aspergillus_Species_in_Quebec_Canada))  **Hamilton**: Eta Ebasi Ashu., et al., 2017 ([Limited evidence of fungicide-driven triazole-resistant Aspergillus fumigatus in Hamilton, Canada](https://cdnsciencepub.com/doi/abs/10.1139/cjm-2017-0410)) | Eta Ebasi Ashu., et al., 2017 ([Limited evidence of fungicide-driven triazole-resistant Aspergillus fumigatus in Hamilton, Canada](https://cdnsciencepub.com/doi/abs/10.1139/cjm-2017-0410)) | CDC., 2024 ([Antimicrobial-Resistant Aspergillus \| Aspergillosis \| CDC](https://www.cdc.gov/aspergillosis/php/guidance/?CDC_AAref_Val=https://www.cdc.gov/fungal/diseases/aspergillosis/antifungal-resistant.html)) | Matthew Cheng., et al., 2019 ([(PDF) Triazole Antifungal Susceptibility Patterns among Aspergillus Species in Québec, Canada](https://www.researchgate.net/publication/332030387_Triazole_Antifungal_Susceptibility_Patterns_among_Aspergillus_Species_in_Quebec_Canada)) |
| Multi-drug resistant Mycobacterium tuberculosis | CNISP-CARSS., 2023 ([Antimicrobial resistance: Seasonal update — Canada.ca](https://health-infobase.canada.ca/carss/amr/results.html?ind=11)) | Elizabeth R Andrews., et al., 2020 ([Underutilization of nontuberculous mycobacterial drug susceptibility testing in Ontario, Canada, 2010–2015 - PMC](https://pmc.ncbi.nlm.nih.gov/articles/PMC9602888/)) | PHAC., 2024, ([Tuberculosis (TB): Prevention and risks - Canada.ca](https://www.canada.ca/en/public-health/services/diseases/tuberculosis/prevention-risks.html#a1)) | PHAC., 2024, ([Tuberculosis in Canada - 2008-2018 Data - Open Government Portal](https://open.canada.ca/data/en/dataset/4dbb9bff-022d-4aab-a11d-0a2e1b0afaad))  Data from 2008-2018 | PHAC., 2024., ([Tuberculosis (TB): Symptoms and treatment - Canada.ca](https://www.canada.ca/en/public-health/services/diseases/tuberculosis.html)) |
| Drug-resistant *Shigella* spp. | PHO., 2023 ([Shigella Antimicrobial Resistance](https://www.publichealthontario.ca/-/media/Documents/S/2023/shigella-antimicrobial-resistance.pdf?rev=b8f50ab8d87e4cbfad1c55e44373909b&sc_lang=en)) | PHO., 2023 ([Shigella Antimicrobial Resistance](https://www.publichealthontario.ca/-/media/Documents/S/2023/shigella-antimicrobial-resistance.pdf?rev=b8f50ab8d87e4cbfad1c55e44373909b&sc_lang=en)) | PHO., 2023, ([Shigella Antimicrobial Resistance](https://www.publichealthontario.ca/-/media/Documents/S/2023/shigella-antimicrobial-resistance.pdf?rev=b8f50ab8d87e4cbfad1c55e44373909b&sc_lang=en)) | PHO., 2023, ([Shigella Antimicrobial Resistance](https://www.publichealthontario.ca/-/media/Documents/S/2023/shigella-antimicrobial-resistance.pdf?rev=b8f50ab8d87e4cbfad1c55e44373909b&sc_lang=en)) | PHO., 2023, ([Shigella Antimicrobial Resistance](https://www.publichealthontario.ca/-/media/Documents/S/2023/shigella-antimicrobial-resistance.pdf?rev=b8f50ab8d87e4cbfad1c55e44373909b&sc_lang=en)) |
| Drug-resistant *Haemophilus influenzae* | Ulanova, M., et al., 2023 ([Epidemiology of invasive Haemophilus influenzae disease in northwestern Ontario: comparison of invasive and noninvasive H. influenzae clinical isolates](https://cdnsciencepub.com/doi/abs/10.1139/cjm-2022-0208?journalCode=cjm#sec-2)) | Ulanova, M., et al., 2023 ([Epidemiology of invasive Haemophilus influenzae disease in northwestern Ontario: comparison of invasive and noninvasive H. influenzae clinical isolates](https://cdnsciencepub.com/doi/abs/10.1139/cjm-2022-0208?journalCode=cjm#sec-2)) | PHAC., 2024 ([Pathogen Safety Data Sheets: Infectious Substances – Haemophilus influenzae (type b) - Canada.ca](https://www.canada.ca/en/public-health/services/laboratory-biosafety-biosecurity/pathogen-safety-data-sheets-risk-assessment/haemophilus-influenzae.html)) | CDC., 2024 ([Chapter 8: Haemophilus influenzae \| Pink Book \| CDC](https://www.cdc.gov/pinkbook/hcp/table-of-contents/chapter-8-haemophilus-influenzae.html?CDC_AAref_Val=https://www.cdc.gov/vaccines/pubs/pinkbook/hib.html)) | PHAC., 2023., ([Haemophilus influenzae disease - Canada.ca](https://www.canada.ca/en/public-health/services/immunization/vaccine-preventable-diseases/haemophilus-influenzae-disease/health-professionals.html)) |
| Carbapenem-resistant *Pseudomonas aeruginosa* | CARA., 2021 ([CARA: Canadian Antimicrobial Resistance Alliance](http://can-r.com/study.php?study=cans2021&year=2021)) | CARA., 2021 ([CARA: Canadian Antimicrobial Resistance Alliance](http://can-r.com/study.php?study=cans2021&year=2021)) | Melissa G McCracken., et al., 2019 ([Characterization of carbapenem-resistant and XDR Pseudomonas aeruginosa in Canada: results of the CANWARD 2007–16 study \| Journal of Antimicrobial Chemotherapy \| Oxford Academic](https://academic.oup.com/jac/article/74/Supplement_4/iv32/5553079?login=false)) | Buehrle DJ., et al., 2016 ([Carbapenem-Resistant Pseudomonas aeruginosa Bacteremia: Risk Factors for Mortality and Microbiologic Treatment Failure \| Antimicrobial Agents and Chemotherapy](https://journals.asm.org/doi/10.1128/aac.01243-16#:~:text=Despite%20the%20use%20of%20treatment%20regimens%20that%20were,and%2030%25%20at%2014%20and%2030%20days%2C%20respectively.)) | PHAC., 2012., [Pathogen Safety Data Sheets: Infectious Substances – Pseudomonas spp. - Canada.ca](https://www.canada.ca/en/public-health/services/laboratory-biosafety-biosecurity/pathogen-safety-data-sheets-risk-assessment/pseudomonas.html) |
| Methicilin-Resistant *Staphylococcus aureus* | CNISP., 2023 ([ccdrv49i05a09-eng.pdf](https://www.canada.ca/content/dam/phac-aspc/documents/services/reports-publications/canada-communicable-disease-report-ccdr/monthly-issue/2023-49/issue-5-may-2023/ccdrv49i05a09-eng.pdf)) | CNISP., 2023 ([ccdrv49i05a09-eng.pdf](https://www.canada.ca/content/dam/phac-aspc/documents/services/reports-publications/canada-communicable-disease-report-ccdr/monthly-issue/2023-49/issue-5-may-2023/ccdrv49i05a09-eng.pdf)) | CNISP., 2023 ([ccdrv49i05a09-eng.pdf](https://www.canada.ca/content/dam/phac-aspc/documents/services/reports-publications/canada-communicable-disease-report-ccdr/monthly-issue/2023-49/issue-5-may-2023/ccdrv49i05a09-eng.pdf)) | CNISP., 2023 ([ccdrv49i05a09-eng.pdf](https://www.canada.ca/content/dam/phac-aspc/documents/services/reports-publications/canada-communicable-disease-report-ccdr/monthly-issue/2023-49/issue-5-may-2023/ccdrv49i05a09-eng.pdf)) | CNISP., 2023 ([ccdrv49i05a09-eng.pdf](https://www.canada.ca/content/dam/phac-aspc/documents/services/reports-publications/canada-communicable-disease-report-ccdr/monthly-issue/2023-49/issue-5-may-2023/ccdrv49i05a09-eng.pdf)) |
| Drug-resistant Invasive Group B Streptococcus | **2017:** Walter H.B. Demczuk., et al., 2017., ([National Surveillance of Antimicrobial Susceptibilities of Neisseria gonorrhoeae](https://publications.gc.ca/collections/collection_2021/aspc-phac/HP57-4-2017-eng.pdf))  **2018:** NLS., 2018 ([National laboratory surveillance of Invasive streptococcal disease in Canada - Annual summary 2018 - Canada.ca](https://www.canada.ca/en/public-health/services/publications/drugs-health-products/national-laboratory-surveillance-invasive-streptococcal-disease-canada-annual-summary-2018.html))  **2019:** NLS., 2019 ([National laboratory surveillance of invasive streptococcal disease in Canada - Annual summary 2019 - Canada.ca](https://www.canada.ca/en/public-health/services/publications/drugs-health-products/national-laboratory-surveillance-invasive-streptococcal-disease-canada-annual-summary-2019.html)) | **2017:** Walter H.B. Demczuk., et al., 2017., ([National Surveillance of Antimicrobial Susceptibilities of Neisseria gonorrhoeae](https://publications.gc.ca/collections/collection_2021/aspc-phac/HP57-4-2017-eng.pdf))  **2018:** NLS., 2018 ([National laboratory surveillance of Invasive streptococcal disease in Canada - Annual summary 2018 - Canada.ca](https://www.canada.ca/en/public-health/services/publications/drugs-health-products/national-laboratory-surveillance-invasive-streptococcal-disease-canada-annual-summary-2018.html))  **2019:** NLS., 2019 ([National laboratory surveillance of invasive streptococcal disease in Canada - Annual summary 2019 - Canada.ca](https://www.canada.ca/en/public-health/services/publications/drugs-health-products/national-laboratory-surveillance-invasive-streptococcal-disease-canada-annual-summary-2019.html)) | PHAC., 2011, ([Pathogen Safety Data Sheets: Infectious Substances – Streptococcus agalactiae - Canada.ca](https://www.canada.ca/en/public-health/services/laboratory-biosafety-biosecurity/pathogen-safety-data-sheets-risk-assessment/streptococcus-agalactiae.html)) | Ikebe T, et al., 2023 ([Serotype Distribution and Antimicrobial Resistance of Streptococcus agalactiae Isolates in Nonpregnant Adults with Streptococcal Toxic Shock Syndrome in Japan in 2014 to 2021 - PMC](https://pmc.ncbi.nlm.nih.gov/articles/PMC10100893/)) | PHAC., 2012., ([Pathogen Safety Data Sheets: Infectious Substances – Streptococcus agalactiae - Canada.ca](https://www.canada.ca/en/public-health/services/laboratory-biosafety-biosecurity/pathogen-safety-data-sheets-risk-assessment/streptococcus-agalactiae.html)) |
| Carbapenem-resistant *Acinetobacter spp.* | CNISP., 2023 ([ccdrv49i05a09s-eng.pdf](https://www.canada.ca/content/dam/phac-aspc/documents/services/reports-publications/canada-communicable-disease-report-ccdr/monthly-issue/2023-49/issue-5-may-2023/ccdrv49i05a09s-eng.pdf)) | CNISP., 2023 ([ccdrv49i05a09s-eng.pdf](https://www.canada.ca/content/dam/phac-aspc/documents/services/reports-publications/canada-communicable-disease-report-ccdr/monthly-issue/2023-49/issue-5-may-2023/ccdrv49i05a09s-eng.pdf)) | CNISP., 2023 ([ccdrv49i05a09s-eng.pdf](https://www.canada.ca/content/dam/phac-aspc/documents/services/reports-publications/canada-communicable-disease-report-ccdr/monthly-issue/2023-49/issue-5-may-2023/ccdrv49i05a09s-eng.pdf)) | Jale Boral., et al, 2023, ([The association between Acinetobacter baumannii infections and the COVID-19 pandemic in an intensive care unit \| Scientific Reports](https://www.nature.com/articles/s41598-022-25493-8#Sec10)) | Jale Boral., et al, 2023, ([The association between Acinetobacter baumannii infections and the COVID-19 pandemic in an intensive care unit \| Scientific Reports](https://www.nature.com/articles/s41598-022-25493-8#Sec10) |
| Drug-resistant *Bacteroides* spp. | James A Karlowsky., et al., 2012 ([Prevalence of Antimicrobial Resistance among Clinical Isolates of Bacteroides fragilis Group in Canada in 2010-2011: CANWARD Surveillance Study - PMC](https://pmc.ncbi.nlm.nih.gov/articles/PMC3294939/)) | James A Karlowsky., et al., 2012 ([Prevalence of Antimicrobial Resistance among Clinical Isolates of Bacteroides fragilis Group in Canada in 2010-2011: CANWARD Surveillance Study - PMC](https://pmc.ncbi.nlm.nih.gov/articles/PMC3294939/)) | PHAC., 2010 ([Pathogen Safety Data Sheets: Infectious Substances – Bacteroides spp. - Canada.ca](https://www.canada.ca/en/public-health/services/laboratory-biosafety-biosecurity/pathogen-safety-data-sheets-risk-assessment/bacteroides.html))  **Non Canadian:** Seyedesomaye Jasemi., et al, 2021 ([Antibiotic resistance pattern of Bacteroides fragilis isolated from clinical and colorectal specimens - PMC](https://pmc.ncbi.nlm.nih.gov/articles/PMC8066845/)) | Luc Dubreuil., et al., 2021 ([Correlation between antibiotic resistance and clinical outcome of anaerobic infections; mini-review - ScienceDirect](https://www.sciencedirect.com/science/article/pii/S1075996421001463#:~:text=The%20mortality%20rate%2C%20for%20patients,isolate%20was%20susceptible%20(16%25).)) | PHAC., 2011., ([Pathogen Safety Data Sheets: Infectious Substances – Bacteroides spp. - Canada.ca](https://www.canada.ca/en/public-health/services/laboratory-biosafety-biosecurity/pathogen-safety-data-sheets-risk-assessment/bacteroides.html)) |
| Drug-resistant Salmonella spp. (Typhoidal) | **CIPARS-CFEZID (Internal)**  – reader must request directly from program area | **CIPARS-CFEZID (Internal)**  – reader must request directly from program area | **2017- pg.100**: CIPARS., 2017 ([Microsoft Word - CIPARS-2017-Figures&tables-EN-(Final)-2019-10-24.docx](https://publications.gc.ca/collections/collection_2019/aspc-phac/HP2-4-2017-4-eng.pdf) )  **2018-pg.108**: CIPARS., 2018 ([HP2-4-2018-eng-4.pdf](https://publications.gc.ca/collections/collection_2020/aspc-phac/HP2-4-2018-eng-4.pdf))  **2019- p.118:** CIPARS, 2019 [HP2-4-2019-eng-4.pdf](https://publications.gc.ca/collections/collection_2022/aspc-phac/HP2-4-2019-eng-4.pdf)  **2020-21- p.5:** CIPARS, 2020-2021 ([canadian-integrated-program-antimicrobial-resistance-surveillance-2022-executive-summary-en.pdf](https://www.canada.ca/content/dam/phac-aspc/documents/services/publications/drugs-health-products-canadian-integrated-program-antimicrobial-resistance-surveillance-2022-executive-summary/canadian-integrated-program-antimicrobial-resistance-surveillance-2022-executive-summary-en.pdf)) | PAHO, WHO., 2018, ([10 October 2018: Salmonella enterica serovar Typhi – Epidemiological Alert - PAHO/WHO \| Pan American Health Organization](https://www.paho.org/en/news/10-10-2018-10-october-2018-salmonella-enterica-serovar-typhi-epidemiological-alert)) | PHAC., 2023., ([Interim guidance on management of infections with a multidrug-resistant strain of Salmonella Newport - Canada.ca](https://www.canada.ca/en/public-health/services/catmat/interim-guidance-management-infections-multidrug-resistant-strain-salmonella-newport.html)) |
| Extended spectrum B-lactamase-producing Enterobacterales | Ambler Class: [Ambler Classification system of β-lactamases - Streck](https://www.streck.com/blog/ambler-classification-of-%CE%B2-lactamases/)  CNISP Report: not clickable link | Ambler Class: [Ambler Classification system of β-lactamases - Streck](https://www.streck.com/blog/ambler-classification-of-%CE%B2-lactamases/)  CNISP Report: not clickable link | CNISP-CARSS., 2024 ([Antimicrobial resistance: Seasonal update — Canada.ca](https://health-infobase.canada.ca/carss/amr/results.html?ind=09)) | CDC., 2019 ([Antibiotic Resistance Threats in the United States, 2019](https://www.cdc.gov/antimicrobial-resistance/media/pdfs/2019-ar-threats-report-508.pdf?CDC_AAref_Val=https://www.cdc.gov/drugresistance/pdf/threats-report/2019-ar-threats-report-508.pdf)) | James A Karlowsky., et al., 2021 ([ESBL-positive Escherichia coli and Klebsiella pneumoniae isolates from across Canada: CANWARD surveillance study, 2007–18 \| Journal of Antimicrobial Chemotherapy \| Oxford Academic](https://academic.oup.com/jac/article/76/11/2815/6347675?login=true)) |
| Drug-resistant *Campylobacter* spp. | CIPARS sentinal sites RAW DATA | CIPARS sentinal sites RAW DATA | PHAC., 2018 ([For health professionals treating campylobacteriosis (Campylobacter) - Canada.ca](https://www.canada.ca/en/public-health/services/diseases/campylobacteriosis-campylobacter/for-health-professionals.html#a2)) | Simon JG., et al., 2020 ([Antimicrobial Resistance of Human Campylobacter Species Infections in Saskatchewan, Canada (1999–2006): A Historical Provincial Collection of All Reported Cases \| Foodborne Pathogens and Disease](https://www.liebertpub.com/doi/10.1089/fpd.2019.2707)) | PHAC., 2018., ([For health professionals treating campylobacteriosis (Campylobacter) - Canada.ca](https://www.canada.ca/en/public-health/services/diseases/campylobacteriosis-campylobacter/for-health-professionals.html#a2)) |
| Drug-resistant Pulmonary nontuberculosis *Mycobacteria* | Elizabeth R Andrews., et al., 2020 ([Underutilization of nontuberculous mycobacterial drug susceptibility testing in Ontario, Canada, 2010–2015 - PMC](https://pmc.ncbi.nlm.nih.gov/articles/PMC9602888/)) | Elizabeth R Andrews., et al., 2020 ([Underutilization of nontuberculous mycobacterial drug susceptibility testing in Ontario, Canada, 2010–2015 - PMC](https://pmc.ncbi.nlm.nih.gov/articles/PMC9602888/)) | PHAC., 2025 ([Canadian Tuberculosis Standards, 8th edition (2022) - Canada.ca](https://www.canada.ca/en/public-health/services/diseases/tuberculosis/health-professionals/canadian-tuberculosis-standards.html)) | PHAC., 2025 ([Canadian Tuberculosis Standards, 8th edition (2022) - Canada.ca](https://www.canada.ca/en/public-health/services/diseases/tuberculosis/health-professionals/canadian-tuberculosis-standards.html)) | PHAC., 2025 ([Canadian Tuberculosis Standards, 8th edition (2022) - Canada.ca](https://www.canada.ca/en/public-health/services/diseases/tuberculosis/health-professionals/canadian-tuberculosis-standards.html)) |
| Vancomycin-resistant *Enterococcus spp.* | CNISP., 2023, ([ccdrv49i05a09-eng.pdf](https://www.canada.ca/content/dam/phac-aspc/documents/services/reports-publications/canada-communicable-disease-report-ccdr/monthly-issue/2023-49/issue-5-may-2023/ccdrv49i05a09-eng.pdf)) | CARSS., 2023, ([Antimicrobial resistance: Seasonal update — Canada.ca](https://health-infobase.canada.ca/carss/amr/)) | CNISP., 2023 ([ccdrv49i05a09-eng.pdf](https://www.canada.ca/content/dam/phac-aspc/documents/services/reports-publications/canada-communicable-disease-report-ccdr/monthly-issue/2023-49/issue-5-may-2023/ccdrv49i05a09-eng.pdf)) | CARSS, CNISP., 2024 ([Antimicrobial resistance: Seasonal update — Canada.ca](https://health-infobase.canada.ca/carss/amr/results.html?ind=10)) | CARS., 2024 ([Antimicrobial resistance: Seasonal update — Canada.ca](https://health-infobase.canada.ca/carss/amr/results.html?ind=10)) |
| Drug-resistant Human immunodeficiency virus | G. Rocheleau., et al., 2018 ([Longitudinal trends of HIV drug resistance in a large Canadian cohort, 1996–2016 - Clinical Microbiology and Infection](https://www.clinicalmicrobiologyandinfection.com/article/S1198-743X(17)30333-6/fulltext)) | G. Rocheleau., et al., 2018 ([Longitudinal trends of HIV drug resistance in a large Canadian cohort, 1996–2016 - Clinical Microbiology and Infection](https://www.clinicalmicrobiologyandinfection.com/article/S1198-743X(17)30333-6/fulltext)) | PHAC., 2022 ([Estimates of HIV incidence, prevalence and Canada’s progress on meeting the 90-90-90 HIV targets, 2020 - Canada.ca](https://www.canada.ca/en/public-health/services/publications/diseases-conditions/estimates-hiv-incidence-prevalence-canada-meeting-90-90-90-targets-2020.html)) | Tianhao Zhang., et al., 2021 ([Relationship Between Drug Resistance and Death in HIV-Infected Patients Receiving Antiretroviral Therapy — 7 PLADs, China, 2010−2019 - PMC](https://pmc.ncbi.nlm.nih.gov/articles/PMC8393015/#:~:text=Among%20HIV-infected%20patients%20receiving,neither%20viral%20load%20nor%20drug)) | PHAC., 2025., ([HIV and AIDS: Symptoms and treatment - Canada.ca](https://www.canada.ca/en/public-health/services/diseases/hiv-aids.html)) |
| *Mycoplasma genitalium* | Anne-Sophie Lê., et al., 2023 ([ccdrv49i1112a03-eng.pdf](https://www.canada.ca/content/dam/phac-aspc/documents/services/reports-publications/canada-communicable-disease-report-ccdr/monthly-issue/2023-49/issue-11-12-november-december-2023/ccdrv49i1112a03-eng.pdf)) | Anne-Sophie Lê., et al., 2023 ([ccdrv49i1112a03-eng.pdf](https://www.canada.ca/content/dam/phac-aspc/documents/services/reports-publications/canada-communicable-disease-report-ccdr/monthly-issue/2023-49/issue-11-12-november-december-2023/ccdrv49i1112a03-eng.pdf)) | PHAC., 2021, ([Mycoplasma Genitalium: Risk factors and clinical manifestation - Canada.ca](https://www.canada.ca/en/public-health/services/infectious-diseases/sexual-health-sexually-transmitted-infections/canadian-guidelines/mycoplasma-genitalium/risk-factors-clinical-manifestation.html#Transmission)) | Anne-Sophie Lê., et al., 2023 ([ccdrv49i1112a03-eng.pdf](https://www.canada.ca/content/dam/phac-aspc/documents/services/reports-publications/canada-communicable-disease-report-ccdr/monthly-issue/2023-49/issue-11-12-november-december-2023/ccdrv49i1112a03-eng.pdf)) | PHAC., 2021., ([Mycoplasma Genitalium: Risk factors and clinical manifestation - Canada.ca](https://www.canada.ca/en/public-health/services/infectious-diseases/sexual-health-sexually-transmitted-infections/canadian-guidelines/mycoplasma-genitalium/risk-factors-clinical-manifestation.html)) |
| Drug-resistant Salmonella spp. (Non-typhoidal) | **CIPARS-CFEZID (Internal)**  – reader must request directly from program area | **CIPARS-CFEZID (Internal)**  – reader must request directly from program area | **2017- pg.100**: CIPARS., 2017 ([Microsoft Word - CIPARS-2017-Figures&tables-EN-(Final)-2019-10-24.docx](https://publications.gc.ca/collections/collection_2019/aspc-phac/HP2-4-2017-4-eng.pdf) )  **2018-pg.108**: CIPARS., 2018 ([HP2-4-2018-eng-4.pdf](https://publications.gc.ca/collections/collection_2020/aspc-phac/HP2-4-2018-eng-4.pdf))  **2019- p.118:** CIPARS, 2019 [HP2-4-2019-eng-4.pdf](https://publications.gc.ca/collections/collection_2022/aspc-phac/HP2-4-2019-eng-4.pdf)  **2020-21- p.5:** CIPARS, 2020-2021 ([canadian-integrated-program-antimicrobial-resistance-surveillance-2022-executive-summary-en.pdf](https://www.canada.ca/content/dam/phac-aspc/documents/services/publications/drugs-health-products-canadian-integrated-program-antimicrobial-resistance-surveillance-2022-executive-summary/canadian-integrated-program-antimicrobial-resistance-surveillance-2022-executive-summary-en.pdf)) | Morten Helms., et al., 2002 ([Excess Mortality Associated with Antimicrobial Drug-Resistant Salmonella Typhimurium - PMC](https://pmc.ncbi.nlm.nih.gov/articles/PMC2732497/#:~:text=Two%20studies%20based%20on%20outbreaks,after%206%20months%20of%20infection).)) | PHAC., 2023., ([Interim guidance on management of infections with a multidrug-resistant strain of Salmonella Newport - Canada.ca](https://www.canada.ca/en/public-health/services/catmat/interim-guidance-management-infections-multidrug-resistant-strain-salmonella-newport.html)) |
| ESBL-Salmonella | CIPARS., 2022 ([canadian-integrated-program-antimicrobial-resistance-surveillance-2022-executive-summary-en.pdf](https://www.canada.ca/content/dam/phac-aspc/documents/services/publications/drugs-health-products-canadian-integrated-program-antimicrobial-resistance-surveillance-2022-executive-summary/canadian-integrated-program-antimicrobial-resistance-surveillance-2022-executive-summary-en.pdf)) | CIPARS., 2022 ([canadian-integrated-program-antimicrobial-resistance-surveillance-2022-executive-summary-en.pdf](https://www.canada.ca/content/dam/phac-aspc/documents/services/publications/drugs-health-products-canadian-integrated-program-antimicrobial-resistance-surveillance-2022-executive-summary/canadian-integrated-program-antimicrobial-resistance-surveillance-2022-executive-summary-en.pdf)) | **2017- pg.100**: CIPARS., 2017 ([Microsoft Word - CIPARS-2017-Figures&tables-EN-(Final)-2019-10-24.docx](https://publications.gc.ca/collections/collection_2019/aspc-phac/HP2-4-2017-4-eng.pdf) )  **2018-pg.108**: CIPARS., 2018 ([HP2-4-2018-eng-4.pdf](https://publications.gc.ca/collections/collection_2020/aspc-phac/HP2-4-2018-eng-4.pdf))  **2019- p.118:** CIPARS, 2019 [HP2-4-2019-eng-4.pdf](https://publications.gc.ca/collections/collection_2022/aspc-phac/HP2-4-2019-eng-4.pdf)  **2020-21- p.5:** CIPARS, 2020-2021 ([canadian-integrated-program-antimicrobial-resistance-surveillance-2022-executive-summary-en.pdf](https://www.canada.ca/content/dam/phac-aspc/documents/services/publications/drugs-health-products-canadian-integrated-program-antimicrobial-resistance-surveillance-2022-executive-summary/canadian-integrated-program-antimicrobial-resistance-surveillance-2022-executive-summary-en.pdf)) | Morten Helms., et al., 2002 ([Excess Mortality Associated with Antimicrobial Drug-Resistant Salmonella Typhimurium - PMC](https://pmc.ncbi.nlm.nih.gov/articles/PMC2732497/#:~:text=Two%20studies%20based%20on%20outbreaks,after%206%20months%20of%20infection).)) | PHAC., 2023., ([Interim guidance on management of infections with a multidrug-resistant strain of Salmonella Newport - Canada.ca](https://www.canada.ca/en/public-health/services/catmat/interim-guidance-management-infections-multidrug-resistant-strain-salmonella-newport.html) |
| Drug-resistant *Influenza* A | **2016-2017:** [fluwatch-2016-2017-51-52-surveillance-influenza-eng.pdf](https://www.canada.ca/content/dam/hc-sc/healthy-canadians/migration/publications/diseases-conditions-maladies-affections/fluwatch-2016-2017-51-52-surveillance-influenza/alt/fluwatch-2016-2017-51-52-surveillance-influenza-eng.pdf)  **2017-2018:** [Fluwatch - December 17 to 30, 2017 (Weeks 51 and 52)](https://www.canada.ca/content/dam/phac-aspc/documents/services/publications/diseases-conditions/fluwatch/2017-2018/weeks51-52-december-17-30-2017/weeks51-52-december-17-30-2017.pdf)  **2018-2019:** [Fluwatch - December 2 to 8, 2018 (Week 49)](https://www.canada.ca/content/dam/phac-aspc/documents/services/publications/diseases-conditions/fluwatch/2018-2019/week51-52-december-16-december-29-2018/week51-52-december-16-december-29-2018.pdf)  **2019-2020**: [pub-eng](https://www.canada.ca/content/dam/phac-aspc/documents/services/publications/diseases-conditions/fluwatch/2019-2020/week51/pub-eng.pdf)  **2020-2021:** [Guidance: Infection Prevention and Control Measures for Healthcare Workers in Acute Care Facilities](https://www.canada.ca/content/dam/phac-aspc/documents/services/publications/diseases-conditions/fluwatch/2020-2021/fw-weeks51-53-2021.pdf)  **2021-2022**: [FluWatch Report ENG](https://www.canada.ca/content/dam/phac-aspc/documents/services/publications/diseases-conditions/fluwatch/2021-2022/fw-weeks50-52-2021-en.pdf) | **2016-2017:** [fluwatch-2016-2017-51-52-surveillance-influenza-eng.pdf](https://www.canada.ca/content/dam/hc-sc/healthy-canadians/migration/publications/diseases-conditions-maladies-affections/fluwatch-2016-2017-51-52-surveillance-influenza/alt/fluwatch-2016-2017-51-52-surveillance-influenza-eng.pdf)  **2017-2018:** [Fluwatch - December 17 to 30, 2017 (Weeks 51 and 52)](https://www.canada.ca/content/dam/phac-aspc/documents/services/publications/diseases-conditions/fluwatch/2017-2018/weeks51-52-december-17-30-2017/weeks51-52-december-17-30-2017.pdf)  **2018-2019:** [Fluwatch - December 2 to 8, 2018 (Week 49)](https://www.canada.ca/content/dam/phac-aspc/documents/services/publications/diseases-conditions/fluwatch/2018-2019/week51-52-december-16-december-29-2018/week51-52-december-16-december-29-2018.pdf)  **2019-2020:** [pub-eng](https://www.canada.ca/content/dam/phac-aspc/documents/services/publications/diseases-conditions/fluwatch/2019-2020/week51/pub-eng.pdf)  **2020-2021:** [Guidance: Infection Prevention and Control Measures for Healthcare Workers in Acute Care Facilities](https://www.canada.ca/content/dam/phac-aspc/documents/services/publications/diseases-conditions/fluwatch/2020-2021/fw-weeks51-53-2021.pdf)  **2021-2022:** [FluWatch Report ENG](https://www.canada.ca/content/dam/phac-aspc/documents/services/publications/diseases-conditions/fluwatch/2021-2022/fw-weeks50-52-2021-en.pdf) | PHAC., 2024 ([Flu (influenza): For health professionals - Canada.ca](https://www.canada.ca/en/public-health/services/diseases/flu-influenza/health-professionals.html)) | Nila J. Dharan., et al., 2009 ([Infections With Oseltamivir-Resistant Influenza A(H1N1) Virus in the United States \| Infectious Diseases \| JAMA \| JAMA Network](https://jamanetwork.com/journals/jama/fullarticle/183543)) | PHAC., 2025., ([Flu (influenza): Symptoms and treatment - Canada.ca](https://www.canada.ca/en/public-health/services/diseases/flu-influenza.html)) |
| *Ureaplasma  spp.* | Subject Matter Expert (Irene Martin)  NML– reader must request directly from program area | Canada is not currently set up for AMR testing per Subject Matter Expert | PHAC., 2011 ([Pathogen Safety Data Sheets: Infectious Substances – Ureaplasma urealyticum - Canada.ca](https://www.canada.ca/en/public-health/services/laboratory-biosafety-biosecurity/pathogen-safety-data-sheets-risk-assessment/ureaplasma-urealyticum.html)) | Charlotte Wigston., et al., 2023 ([Mycoplasma and Ureaplasma Donor-Derived Infection and Hyperammonemia Syndrome in 4 Solid Organ Transplant Recipients From a Single Donor \| Open Forum Infectious Diseases \| Oxford Academic](https://academic.oup.com/ofid/article/10/6/ofad263/7174013)) | PHAC., 2012., ([Pathogen Safety Data Sheets: Infectious Substances – Ureaplasma urealyticum - Canada.ca](https://www.canada.ca/en/public-health/services/laboratory-biosafety-biosecurity/pathogen-safety-data-sheets-risk-assessment/ureaplasma-urealyticum.html)) |
| *Clostridium difficile* | CNISP., 2023. ([ccdrv49i05a09-eng.pdf](https://www.canada.ca/content/dam/phac-aspc/documents/services/reports-publications/canada-communicable-disease-report-ccdr/monthly-issue/2023-49/issue-5-may-2023/ccdrv49i05a09-eng.pdf)) | CARSS., 2023 ([Canadian Antimicrobial Resistance Surveillance System (CARSS) 2023 executive summary - Canada.ca](https://www.canada.ca/en/public-health/services/publications/drugs-health-products/canadian-antimicrobial-resistance-surveillance-system-2023-executive-summary.html#a2)) | CARSS., 2023 ([Canadian Antimicrobial Resistance Surveillance System (CARSS) 2023 executive summary - Canada.ca](https://www.canada.ca/en/public-health/services/publications/drugs-health-products/canadian-antimicrobial-resistance-surveillance-system-2023-executive-summary.html#a2)) | CNISP., 2023. ([ccdrv49i05a09-eng.pdf](https://www.canada.ca/content/dam/phac-aspc/documents/services/reports-publications/canada-communicable-disease-report-ccdr/monthly-issue/2023-49/issue-5-may-2023/ccdrv49i05a09-eng.pdf)) | PHAC., 2012., ([C. difficile (Clostridium difficile) - Canada.ca](https://www.canada.ca/en/public-health/services/diseases/c-difficile.html)) |
| Drug -resistant *Candida* spp.* | Jeff Fuller., 2019, ([Species distribution and antifungal susceptibility of invasive Candida isolates from Canadian hospitals: results of the CANWARD 2011–16 study \| Journal of Antimicrobial Chemotherapy \| Oxford Academic](https://academic.oup.com/jac/article/74/Supplement_4/iv48/5553081?login=false)) | Jeff Fuller., 2019, ([Species distribution and antifungal susceptibility of invasive Candida isolates from Canadian hospitals: results of the CANWARD 2011–16 study \| Journal of Antimicrobial Chemotherapy \| Oxford Academic](https://academic.oup.com/jac/article/74/Supplement_4/iv48/5553081?login=false)) | PHAC., 2014 ([Pathogen Safety Data Sheets: Infectious Substances – Candida albicans - Canada.ca](https://www.canada.ca/en/public-health/services/laboratory-biosafety-biosecurity/pathogen-safety-data-sheets-risk-assessment/candida-albicans-pathogen-safety-data-sheet.html)) | CDC., 2019, ([Drug-Resistant Candida Species](https://www.cdc.gov/antimicrobial-resistance/media/pdfs/candida-508.pdf?CDC_AAref_Val=https://www.cdc.gov/drugresistance/pdf/threats-report/candida-508.pdf)) | PHAC., 2014., ([Pathogen Safety Data Sheets: Infectious Substances – Candida albicans - Canada.ca](https://www.canada.ca/en/public-health/services/laboratory-biosafety-biosecurity/pathogen-safety-data-sheets-risk-assessment/candida-albicans-pathogen-safety-data-sheet.html)) |
| Drug-resistant *Helicobacter pylori* | Thomas Krahn., 2024, ([Success of Helicobacter pylori Guideline-based Treatment of Newly Diagnosed and Previously Treated Patients During 2007–2021 in Edmonton, Alberta \| Journal of the Canadian Association of Gastroenterology \| Oxford Academic](https://academic.oup.com/jcag/article/7/3/221/7473647)) | Thomas Krahn., 2024, ([Success of Helicobacter pylori Guideline-based Treatment of Newly Diagnosed and Previously Treated Patients During 2007–2021 in Edmonton, Alberta \| Journal of the Canadian Association of Gastroenterology \| Oxford Academic](https://academic.oup.com/jcag/article/7/3/221/7473647)) | PHAC., 2024, ([Helicobacter pylori: Infectious substances pathogen safety data sheet - Canada.ca](https://www.canada.ca/en/public-health/services/laboratory-biosafety-biosecurity/pathogen-safety-data-sheets-risk-assessment/helicobacter-pylori.html)) | Brian White., et al., 2022 ([Clinical Factors Implicated in Antibiotic Resistance in Helicobacter pylori Patients - PMC](https://pmc.ncbi.nlm.nih.gov/articles/PMC8876575/)) | PHAC., 2024., ([Helicobacter pylori: Infectious substances pathogen safety data sheet - Canada.ca](https://www.canada.ca/en/public-health/services/laboratory-biosafety-biosecurity/pathogen-safety-data-sheets-risk-assessment/helicobacter-pylori.html)) |
| Drug-resistant *Treponema pallidum* | Shuel, Michelle., 2018, ([Sexually Transmitted Diseases](https://journals.lww.com/stdjournal/abstract/2018/04000/molecular_typing_and_macrolide_resistance_of.4.aspx)) | Shuel, Michelle., 2018, ([Sexually Transmitted Diseases](https://journals.lww.com/stdjournal/abstract/2018/04000/molecular_typing_and_macrolide_resistance_of.4.aspx)) | PHAC., 2024, ([Syphilis: Symptoms and treatment - Canada.ca](https://www.canada.ca/en/public-health/services/diseases/syphilis.html#a2)) | Noel C. Barragan., et al., 2023 ([Current Trends in Syphilis Mortality in the United States, 2015–2020](https://www.mdpi.com/2674-0710/2/2/5)) | PHAC., 2018., ([Archived - Stigma and sexually transmitted infections - Canada.ca](https://www.canada.ca/en/public-health/services/reports-publications/canada-communicable-disease-report-ccdr/monthly-issue/2018-44/issue-2-february-1-2018/article-5-stigma-sexually-transmitted-infections.html)) |
| Drug-resistant Chlamydia trachomatis | PHAC., 2022, ([Chlamydia and LGV guide: Treatment and follow-up - Canada.ca](https://www.canada.ca/en/public-health/services/infectious-diseases/sexual-health-sexually-transmitted-infections/canadian-guidelines/chlamydia-lgv/treatment-follow-up.html)) | PHAC., 2022, ([Chlamydia and LGV guide: Treatment and follow-up - Canada.ca](https://www.canada.ca/en/public-health/services/infectious-diseases/sexual-health-sexually-transmitted-infections/canadian-guidelines/chlamydia-lgv/treatment-follow-up.html)) | PHAC., 2022, ([Chlamydia and LGV guide: Treatment and follow-up - Canada.ca](https://www.canada.ca/en/public-health/services/infectious-diseases/sexual-health-sexually-transmitted-infections/canadian-guidelines/chlamydia-lgv/treatment-follow-up.html)) | Lancet., 2016., ([Global, regional, and national life expectancy, all-cause mortality, and cause-specific mortality for 249 causes of death, 1980–2015: a systematic analysis for the Global Burden of Disease Study 2015 - PMC](https://pmc.ncbi.nlm.nih.gov/articles/PMC5388903/)) | PHAC., 2024., ([Chlamydia and LGV guide: Risk factors and clinical manifestations - Canada.ca](https://www.canada.ca/en/public-health/services/infectious-diseases/sexual-health-sexually-transmitted-infections/canadian-guidelines/chlamydia-lgv/risk-factors-clinical-manifestation.html)) |

| AMR Pathogen | Criteria | | | |
| --- | --- | --- | --- | --- |
|  | 6 | 7 | 8 | 9 |
| *Candida auris* | [C. auris: Infectious Substances pathogen](https://www.canada.ca/en/public-health/services/laboratory-biosafety-biosecurity/pathogen-safety-data-sheets-risk-assessment/candida-auris.html)  but refers to [CDC;](https://www.cdc.gov/candida-auris/hcp/clinical-care/?CDC_AAref_Val=https://www.cdc.gov/fungal/candida-auris/c-auris-treatment.html) [Schwartz & Hammond, 2017](https://pubmed.ncbi.nlm.nih.gov/29770082/);  [PHO, 2019;](https://www.publichealthontario.ca/-/media/Documents/P/2019/pidac-ipac-candida-auris.pdf?rev=7f655451d9144044b38ca13c77649ee3&sc_lang=en)  ARNI (Internal), 2023;  NMLB (Internal) – reader must request directly from program area | [CNISP 2017-2021 reports p.240](https://www.canada.ca/content/dam/phac-aspc/documents/services/reports-publications/canada-communicable-disease-report-ccdr/monthly-issue/2023-49/issue-5-may-2023/ccdrv49i05a09-eng.pdf) | [Candida auris Infection Prevention and Control in Canadian Healthcare Settings - Canada.ca](https://www.canada.ca/en/services/health/publications/diseases-conditions/candida-auris-infection-prevention-control-canadian-healthcare-settings.html#a1) | [Candida auris Infection Prevention and Control in Canadian Healthcare Settings - Canada.ca](https://www.canada.ca/en/services/health/publications/diseases-conditions/candida-auris-infection-prevention-control-canadian-healthcare-settings.html#a1) |
| Carbapenem-resistant Enterobacterales | [German, GJ et al., 2016](https://www.canada.ca/en/public-health/services/reports-publications/canada-communicable-disease-report-ccdr/monthly-issue/2016-42/ccdr-volume-42-4-april-7-2016/ccdr-volume-42-4-april-7-2016-vaccine-preventable-diseases-3.html);  [IDSA, 2024](https://www.idsociety.org/practice-guideline/amr-guidance/#Section3:Carbapenem-ResistantEnterobacterales);  [Trecarichi & Tumbarello, 2017](https://pmc.ncbi.nlm.nih.gov/articles/PMC5477725/%22%20/l%20%22cit0006);  [Kohler, Philipp P et al., 2018](https://pmc.ncbi.nlm.nih.gov/articles/PMC6106407/);  [CNISP 2022,](https://pmc.ncbi.nlm.nih.gov/articles/PMC10718477/#:~:text=Amikacin%2042%2026,4)  NMLB (Internal) – reader must request directly from program area;  [Johnstone, J et al., 2017](https://www.cmaj.ca/content/189/35/E1115) | [CNISP 2017-2021 report p.241](https://www.canada.ca/content/dam/phac-aspc/documents/services/reports-publications/canada-communicable-disease-report-ccdr/monthly-issue/2023-49/issue-5-may-2023/ccdrv49i05a09-eng.pdf) | [CARSS 2023 Report p. 22](https://www.canada.ca/content/dam/phac-aspc/documents/services/publications/drugs-health-products/canadian-antimicrobial-resistance-surveillance-system-report-2022/canadian-antimicrobial-resistance-surveillance-system-report-2022.pdf) | [Guidance: Infection Prevention and Control Measures for Healthcare Workers in All Healthcare Settings - Canada.ca](https://www.canada.ca/en/public-health/services/infectious-diseases/nosocomial-occupational-infections/guidance-infection-prevention-control-measures-healthcare-workers-healthcare-settings.html) |
| Drug-resistant *Neisseria gonorrhoeae* | [PHAC, 2021](https://www.canada.ca/en/public-health/services/infectious-diseases/sexual-health-sexually-transmitted-infections/canadian-guidelines/national-advisory-committee-stbbi/statements/interim-guidance-treatment-uncomplicated-gonococcal-infections.html);  [PHAC, 2024;](https://www.canada.ca/en/public-health/services/infectious-diseases/sexual-health-sexually-transmitted-infections/canadian-guidelines/gonorrhea.html)  [PHO Microbiology Rounds](https://www.publichealthontario.ca/en/Education-and-Events/Events-and-Presentations/2024/10/National-Surveillance-Neisseria-Gonorrhoeae-AMR-Canada?utm_source=chatgpt.com)**,**  [PHAC, 2020;](https://www.canada.ca/en/public-health/services/publications/drugs-health-products/national-surveillance-antimicrobial-susceptibilities-neisseria-gonorrhoeae-annual-summary-2018.html)  [Thorington, Robyn et al., 2022,](https://pmc.ncbi.nlm.nih.gov/articles/PMC10829890/)  [Chow et al., 2024(Australia)]](https://academic.oup.com/jid/article/230/5/e1121/7693754?login=true);  NMLB (Internal) – reader must request directly from program area;  [PHAC, 2020;](https://www.canada.ca/en/public-health/services/publications/drugs-health-products/national-surveillance-antimicrobial-susceptibilities-neisseria-gonorrhoeae-annual-summary-2018.html)  [CARSS, 2024](https://health-infobase.canada.ca/carss/amr/results.html?ind=12) | [Sawatzky P, et al., 2023](https://www.canada.ca/content/dam/phac-aspc/documents/services/reports-publications/canada-communicable-disease-report-ccdr/monthly-issue/2023-49/issue-9-september-2023/ccdrv49i09a05-eng.pdf) | [Gonorrhea guide: Treatment and follow-up - Canada.ca](https://www.canada.ca/en/public-health/services/infectious-diseases/sexual-health-sexually-transmitted-infections/canadian-guidelines/gonorrhea/treatment-follow-up.html)  [Increasing Azithromycin Resistance in Neisseria gonorrhoeae Due to NG-MAST 12302 Clonal Spread in Canada, 2015 to 2018 \| Antimicrobial Agents and Chemotherapy;](https://journals.asm.org/doi/10.1128/aac.01688-21)  [Ceftriaxone-Resistant Neisseria gonorrhoeae, Canada, 2017 - PubMed;](https://pubmed.ncbi.nlm.nih.gov/29131780/)  Antimicrobial susceptibilities of Neisseria gonorrhoeae in Canada, 2020 [[ccdrv48i1112a10-eng.pdf]](https://www.canada.ca/content/dam/phac-aspc/documents/services/reports-publications/canada-communicable-disease-report-ccdr/monthly-issue/2022-48/issue-11-12-november-december-2022/ccdrv48i1112a10-eng.pdf) | [Gonorrhea guide: Prevention and control - Canada.ca](https://www.canada.ca/en/public-health/services/infectious-diseases/sexual-health-sexually-transmitted-infections/canadian-guidelines/gonorrhea/prevention-control.html) |
| Drug-resistant Invasive Group A Streptococcus | [File, 2006](https://www.sciencedirect.com/science/article/pii/S1198743X14613203#:~:text=Certain%20%CE%B2%2Dlactams%20(amoxicillin%2C,that%20remain%20effective%20against%20DRSP.);  [Golden, Alyssa et al., 2022,](https://pmc.ncbi.nlm.nih.gov/articles/PMC10732480/)  [Alford et al., 2023,](https://academic.oup.com/jac/article/78/Supplement_1/i8/7147760)  Z[hanel, Lynch, & Adam, 2023;](https://academic.oup.com/jac/article/78/Supplement_1/i2/7147763)  [CARSS, 2021](https://www.canada.ca/en/public-health/services/publications/drugs-health-products/canadian-antimicrobial-resistance-surveillance-system-report-2021.html#a57) | [CARSS, 2022](https://www.canada.ca/content/dam/phac-aspc/documents/services/publications/drugs-health-products/canadian-antimicrobial-resistance-surveillance-system-report-2022/canadian-antimicrobial-resistance-surveillance-system-report-2022.pdf) | [Golden, Alyssa et al., 2022,](https://pmc.ncbi.nlm.nih.gov/articles/PMC10732480/)  [Invasive group A streptococcal disease surveillance in Canada, 2021-2022 - PubMed;](https://pubmed.ncbi.nlm.nih.gov/38835501/)  [Invasive group A streptococcal disease: Management and chemoprophylaxis \| Canadian Paediatric Society](https://cps.ca/documents/position/Invasive-group-A-streptococcal-disease) | [Group A Streptococcal diseases: Risks and prevention - Canada.ca](https://www.canada.ca/en/public-health/services/diseases/group-a-streptococcal-diseases/risks-prevention.html) |
| Clindamycin-resistant Group A Streptococcus | [Moore et al, 2019](https://cps.ca/documents/position/Invasive-group-A-streptococcal-disease);  [Sauve et al., 2021;](https://cps.ca/documents/position/group-a-streptococcal?utm_source=chatgpt.com)  [Golden et al., 2024; Moore et al, 2019]](https://www.canada.ca/content/dam/phac-aspc/documents/services/reports-publications/canada-communicable-disease-report-ccdr/monthly-issue/2024-50/issue-5-may-2024/ccdrv50i05a03-eng.pdf) | [Moore et al, 2019](https://cps.ca/documents/position/Invasive-group-A-streptococcal-disease);  [Sauve et al., 2021;](https://cps.ca/documents/position/group-a-streptococcal?utm_source=chatgpt.com)  [Golden et al., 2024; Moore et al, 2019]](https://www.canada.ca/content/dam/phac-aspc/documents/services/reports-publications/canada-communicable-disease-report-ccdr/monthly-issue/2024-50/issue-5-may-2024/ccdrv50i05a03-eng.pdf) | [Moore et al, 2019](https://cps.ca/documents/position/Invasive-group-A-streptococcal-disease);  [Sauve et al., 2021;](https://cps.ca/documents/position/group-a-streptococcal?utm_source=chatgpt.com)  [Golden et al., 2024; Moore et al, 2019]](https://www.canada.ca/content/dam/phac-aspc/documents/services/reports-publications/canada-communicable-disease-report-ccdr/monthly-issue/2024-50/issue-5-may-2024/ccdrv50i05a03-eng.pdf) | [Moore et al, 2019](https://cps.ca/documents/position/Invasive-group-A-streptococcal-disease);  [Sauve et al., 2021;](https://cps.ca/documents/position/group-a-streptococcal?utm_source=chatgpt.com)  [Golden et al., 2024; Moore et al, 2019]](https://www.canada.ca/content/dam/phac-aspc/documents/services/reports-publications/canada-communicable-disease-report-ccdr/monthly-issue/2024-50/issue-5-may-2024/ccdrv50i05a03-eng.pdf) |
| Drug-resistant Streptococcus pneumoniae | [CARSS, 2022](https://www.canada.ca/content/dam/phac-aspc/documents/services/publications/drugs-health-products/canadian-antimicrobial-resistance-surveillance-system-report-2022/canadian-antimicrobial-resistance-surveillance-system-report-2022.pdf) | [CARSS, 2022](https://www.canada.ca/content/dam/phac-aspc/documents/services/publications/drugs-health-products/canadian-antimicrobial-resistance-surveillance-system-report-2022/canadian-antimicrobial-resistance-surveillance-system-report-2022.pdf) | [Epidemiology of invasive pneumococcal disease in indigenous and non-indigenous adults in northwestern Ontario, Canada, 2006–2015 - PMC](https://pmc.ncbi.nlm.nih.gov/articles/PMC6280531/)  [Characterization of MDR and XDR Streptococcus pneumoniae in Canada, 2007-13 - PubMed;](https://pubmed.ncbi.nlm.nih.gov/25921512/)  [Invasive pneumococcal disease surveillance in Canada, 2021-2022 - PubMed;](https://pubmed.ncbi.nlm.nih.gov/38835503/)  [Canadian Antimicrobial Resistance Surveillance System (CARSS) - Canada.ca](https://www.canada.ca/en/public-health/services/publications/drugs-health-products/canadian-antimicrobial-resistance-surveillance-system-2024-executive-summary.html) 2024 | [Recommended immunization schedules: Canadian Immunization Guide - Canada.ca](https://www.canada.ca/en/public-health/services/publications/healthy-living/canadian-immunization-guide-part-1-key-immunization-information/page-13-recommended-immunization-schedules.html) |
| Drug-resistant *Aspergillus* spp. | [IDSA, 2016;](https://pubmed.ncbi.nlm.nih.gov/27365388/)  [Ashu et al., 2017;](https://cdnsciencepub.com/doi/abs/10.1139/cjm-2017-0410?journalCode=cjm)  [Fan, Y., Korfanty, G. A., & Xu, J. (2021)](https://www.mdpi.com/2309-608X/7/10/860) | [Not Notifiable nor reportable in Canada. May be submitted PTs upon request](https://diseases.canada.ca/notifiable/diseases-list) | [(PDF) Triazole Antifungal Susceptibility Patterns among Aspergillus Species in Québec, Canada](https://www.researchgate.net/publication/332030387_Triazole_Antifungal_Susceptibility_Patterns_among_Aspergillus_Species_in_Quebec_Canada) | [Prevalence and mechanisms of azole resistance in clinical isolates of Aspergillus section Fumigati species in a Canadian tertiary care centre, 2000 to 2013;](https://pmc.ncbi.nlm.nih.gov/articles/PMC7069474/pdf/dkz534.pdf)  [Editorial Commentary: Voriconazole Resistance in Aspergillus fumigatus: Should We Be Concerned? \| Clinical Infectious Diseases \| Oxford Academic](https://academic.oup.com/cid/article-abstract/57/4/521/348913?redirectedFrom=fulltext) |
| Multi-drug resistant Mycobacterium tuberculosis | [Canadian Tuberculosis Standards, 2025;](https://manuals.cts-sct.ca/documentation/chapter-8-drug-resistant-tuberculosis/4-management-of-drug-resistant-tb/) [CARSS, 2023;](https://health-infobase.canada.ca/carss/amr/results.html?ind=11) | 2017-2020: CARSS 2022 p.37 2021: CARSS 2023: <https://health-infobase.canada.ca/carss/amr/results.html?ind=11> | [Tuberculosis (TB): Monitoring - Canada.ca](https://www.canada.ca/en/public-health/services/diseases/tuberculosis/surveillance.html)  [Tuberculosis in Canada: Epidemiological update 2022 - Canada.ca;](https://www.canada.ca/en/public-health/services/publications/diseases-conditions/tuberculosis-canada-epidemiological-update-2022.html)  [CCOHS: Multi-Drug Resistant Tuberculosis (MDR-TB);](https://www.ccohs.ca/oshanswers/diseases/mdr_tb.html)  [Canadian Tuberculosis Standards, 8th edition (2022) - Canada.ca;](https://www.canada.ca/en/public-health/services/diseases/tuberculosis/health-professionals/canadian-tuberculosis-standards.html)  [Invasive bacterial diseases in northern Canada, 1999 to 2018;](https://www.canada.ca/content/dam/phac-aspc/documents/services/reports-publications/canada-communicable-disease-report-ccdr/monthly-issue/2021-47/issue-11-november-2021/ccdrv47i11a09-eng.pdf)  [Inuit Tuberculosis Elimination Framework — Inuit Tapiriit Kanatami;](https://www.itk.ca/inuittbeliminationframework/#:~:text=Inuit%20Tuberculosis%20Elimination%20Framework%20Released%20December%2010%2C%202018%E2%80%94,tuberculosis%20%28TB%29%20among%20Inuit%20living%20in%20Inuit%20Nunangat.)  [Directly observed therapy for treating tuberculosis - PMC](https://pmc.ncbi.nlm.nih.gov/articles/PMC4460720/) | [Tuberculosis (TB): For health professionals - Canada.ca](https://www.canada.ca/en/public-health/services/diseases/tuberculosis/health-professionals.html) |
| Drug-resistant *Shigella* spp. | [PHO, 2023](https://www.publichealthontario.ca/-/media/Documents/S/2023/shigella-antimicrobial-resistance.pdf?rev=b8f50ab8d87e4cbfad1c55e44373909b&sc_lang=en) | [PHO 2023 Surveillance Report](https://www.publichealthontario.ca/-/media/Documents/S/2023/shigella-antimicrobial-resistance.pdf?rev=b8f50ab8d87e4cbfad1c55e44373909b&sc_lang=en) | [Multidrug-Resistant Shigella sonnei Bacteremia among Persons Experiencing Homelessness, Vancouver, British Columbia, Canada - PMC](https://pmc.ncbi.nlm.nih.gov/articles/PMC10370870/#:~:text=Increased%20invasive%20bloodstream%20infections%20caused,1.1.)  [Shigella Antimicrobial Resistance](https://www.publichealthontario.ca/-/media/Documents/S/2023/shigella-antimicrobial-resistance.pdf?rev=260ed79450d445dfbc8b53bb91750544&sc_lang=en) (PHO)  [Multidrug-Resistant Shigella sonnei Bacteremia among Persons Experiencing Homelessness, Vancouver, British Columbia, Canada - Volume 29, Number 8—August 2023 - Emerging Infectious Diseases journal - CDC](https://wwwnc.cdc.gov/eid/article/29/8/23-0323_article?utm_source=chatgpt.com) | [For health professionals: Shigellosis (Shigella) - Canada.ca](https://www.canada.ca/en/public-health/services/diseases/shigella/health-professionals.html) |
| Drug-resistant *Haemophilus influenzae* | [Canada.ca/PathogenSafetyDataSheet,2023](https://www.canada.ca/en/public-health/services/laboratory-biosafety-biosecurity/pathogen-safety-data-sheets-risk-assessment/haemophilus-influenzae.html) | [McTaggart L, et al., 2021](https://pmc.ncbi.nlm.nih.gov/articles/PMC8510165/pdf/spectrum.00803-21.pdf) | [Haemophilus influenzae serotype a as a cause of serious invasive infections - ScienceDirect](https://www.sciencedirect.com/science/article/abs/pii/S1473309913701701?via%3Dihub#bib74)  [Increased Incidence of Invasive Haemophilus influenzae Disease Driven by Non-Type B Isolates in Ontario, Canada, 2014 to 2018 - PubMed](https://pubmed.ncbi.nlm.nih.gov/34612671/)  [The Epidemiology of Invasive Haemophilus influenzae Non-Serotype B Disease in Ontario, Canada from 2004 to 2013 - PubMed;](https://pubmed.ncbi.nlm.nih.gov/26569613/)  [Continuing surveillance of invasive Haemophilus influenzae disease in northwestern Ontario emphasizes the importance of serotype a and non-typeable strains as causes of serious disease: a Canadian Immunization Research Network (CIRN) Study - PubMed](https://pubmed.ncbi.nlm.nih.gov/31242396/) | [Haemophilus influenzae disease - Canada.ca](https://www.canada.ca/en/public-health/services/immunization/vaccine-preventable-diseases/haemophilus-influenzae-disease/health-professionals.html) |
| Carbapenem-resistant *Pseudomonas aeruginosa* | [IDSA, 2024;](https://www.idsociety.org/practice-guideline/amr-guidance/#Section4:PseudomonasaeruginosawithDifficult-to-TreatResistance)  [Walkty, A et al., 2013,](https://pmc.ncbi.nlm.nih.gov/articles/PMC3811242/?utm_source=chatgpt.com)  [McCracken MG,, et al.,2019;](https://pubmed.ncbi.nlm.nih.gov/31505643/)  [Karlowsky, James A et al., 2022;](https://pmc.ncbi.nlm.nih.gov/articles/PMC9430561/) | [CANWARD Susceptibility Map](http://can-r.com/study.php?study=cans2021&year=2021) | [Characterization of carbapenem-resistant and XDR Pseudomonas aeruginosa in Canada: results of the CANWARD 2007–16 study \| Journal of Antimicrobial Chemotherapy \| Oxford Academic](https://academic.oup.com/jac/article/74/Supplement_4/iv32/5553079?login=false) | [Guidance: Infection Prevention and Control Measures for Healthcare Workers in All Healthcare Settings - Canada.ca](https://www.canada.ca/en/public-health/services/infectious-diseases/nosocomial-occupational-infections/guidance-infection-prevention-control-measures-healthcare-workers-healthcare-settings.html) |
| Methicilin-Resistant *Staphylococcus aureus* | AMR-Specific Pathogen [[Liu et al, IDSA, 2011](https://academic.oup.com/cid/article/52/3/e18/306145?login=false)]  Syndromic [[Bugs & Drugs, 2016](https://www.bugsanddrugs.org/18AE9616-C3A6-4578-A6D2-8E63045C0D3A)];  [CARSS, 2021](https://www.canada.ca/en/public-health/services/publications/drugs-health-products/canadian-antimicrobial-resistance-surveillance-system-report-2021.html?utm_source=chatgpt.com),  [Kimberly R et al., 2019](https://pmc.ncbi.nlm.nih.gov/articles/PMC6495025/) | [CNISP 2017-2021 report p.238](https://www.canada.ca/content/dam/phac-aspc/documents/services/reports-publications/canada-communicable-disease-report-ccdr/monthly-issue/2023-49/issue-5-may-2023/ccdrv49i05a09-eng.pdf) | [The Prevalence, Risk, and Management of Methicillin-Resistant Staphylococcus aureus Infection in Diverse Populations across Canada: A Systematic Review](https://www.mdpi.com/2076-0817/10/4/393)  [ccdrv48i78a03-eng.pdf](https://www.canada.ca/content/dam/phac-aspc/documents/services/reports-publications/canada-communicable-disease-report-ccdr/monthly-issue/2022-48/issue-7-8-july-august-2022/ccdrv48i78a03-eng.pdf) [CNISP 2022]  [The emergence of methicillin-resistant Staphylococcus aureus as a community-acquired pathogen in Canada - PMC](https://pmc.ncbi.nlm.nih.gov/articles/PMC2094949/) | [Guidance: Infection Prevention and Control Measures for Healthcare Workers in All Healthcare Settings - Canada.ca](https://www.canada.ca/en/public-health/services/infectious-diseases/nosocomial-occupational-infections/guidance-infection-prevention-control-measures-healthcare-workers-healthcare-settings.html) |
| Drug-resistant Invasive Group B Streptococcus | [Heelan et al, 2004;](https://pmc.ncbi.nlm.nih.gov/articles/PMC356858/?utm_source=chatgpt.com)  [National laboratory surveillance of invasive streptococcal disease, 2019,](https://www.canada.ca/en/public-health/services/publications/drugs-health-products/national-laboratory-surveillance-invasive-streptococcal-disease-canada-annual-summary-2019.html?utm_source=chatgpt.com)  [Ma A,Thompson LA, Corsiatto T, Hurteau D, Tyrrell GJ, 2021.](https://doi.org/10.1128/Spectrum.01283-21) | 2017: [National Surveillance of Antimicrobial Susceptibilities of Neisseria gonorrhoeae](https://publications.gc.ca/collections/collection_2021/aspc-phac/HP57-4-2017-eng.pdf)  2018: [National laboratory surveillance of Invasive streptococcal disease in Canada - Annual summary 2018 - Canada.ca](https://www.canada.ca/en/public-health/services/publications/drugs-health-products/national-laboratory-surveillance-invasive-streptococcal-disease-canada-annual-summary-2018.html)  2019: [National laboratory surveillance of invasive streptococcal disease in Canada - Annual summary 2019 - Canada.ca](https://www.canada.ca/en/public-health/services/publications/drugs-health-products/national-laboratory-surveillance-invasive-streptococcal-disease-canada-annual-summary-2019.html) | [Huang G, et al., 2021](https://www.canada.ca/content/dam/phac-aspc/documents/services/reports-publications/canada-communicable-disease-report-ccdr/monthly-issue/2021-47/issue-11-november-2021/ccdrv47i11a09-eng.pdf)  [Correction for Alhhazmi et al., Epidemiology of Invasive Group B Streptococcal Disease in Alberta, Canada, from 2003 to 2013 - PubMed](https://pubmed.ncbi.nlm.nih.gov/28031447/)  [Emergence of Serotype IV Group B Streptococcus Adult Invasive Disease in Manitoba and Saskatchewan, Canada, Is Driven by Clonal Sequence Type 459 Strains - PubMed](https://pubmed.ncbi.nlm.nih.gov/26135871/)  [Invasive group A streptococcal disease surveillance in Canada, 2020, CCDR 48(9) - Canada.ca](https://www.canada.ca/en/public-health/services/reports-publications/canada-communicable-disease-report-ccdr/monthly-issue/2022-48/issue-9-september-2022/invasive-group-a-streptococcal-disease-surveillance-canada-2020.html) | [Guidance: Infection Prevention and Control Measures for Healthcare Workers in All Healthcare Settings - Canada.ca](https://www.canada.ca/en/public-health/services/infectious-diseases/nosocomial-occupational-infections/guidance-infection-prevention-control-measures-healthcare-workers-healthcare-settings.html) |
| Carbapenem-resistant *Acinetobacter spp.* | [German, GJ et al., 2016](https://www.canada.ca/en/public-health/services/reports-publications/canada-communicable-disease-report-ccdr/monthly-issue/2016-42/ccdr-volume-42-4-april-7-2016/ccdr-volume-42-4-april-7-2016-vaccine-preventable-diseases-3.html);  [IDSA, 2024](https://www.idsociety.org/practice-guideline/amr-guidance/#Section5:Carbapenem-ResistantAcinetobacterbaumannii);  [Boyd et al., 2019](https://academic.oup.com/jac/article/74/2/315/5127718);  [Alina et al., 2022;](https://pmc.ncbi.nlm.nih.gov/articles/PMC9040734/)  NMLB (Internal) – reader must request directly from program area | [CNISP 2017-2021 report p.240](https://www.canada.ca/content/dam/phac-aspc/documents/services/reports-publications/canada-communicable-disease-report-ccdr/monthly-issue/2023-49/issue-5-may-2023/ccdrv49i05a09-eng.pdf) | [CNISP 2017-2021 report p.240](https://www.canada.ca/content/dam/phac-aspc/documents/services/reports-publications/canada-communicable-disease-report-ccdr/monthly-issue/2023-49/issue-5-may-2023/ccdrv49i05a09-eng.pdf) | [Guidance: Infection Prevention and Control Measures for Healthcare Workers in All Healthcare Settings – Canada.ca](https://www.canada.ca/en/public-health/services/infectious-diseases/nosocomial-occupational-infections/guidance-infection-prevention-control-measures-healthcare-workers-healthcare-settings.html) |
| Drug-resistant *Bacteroides* spp. | [Jasemi et al.2021](https://ann-clinmicrob.biomedcentral.com/articles/10.1186/s12941-021-00435-w) ;  [Kajihara et al. 2023;](https://pubmed.ncbi.nlm.nih.gov/37469615/)  [Karlowsky et al. 2012](https://journals.asm.org/doi/full/10.1128/aac.05823-11) | [Frobes J, et al., 2021 [PHO Labratory Data]](https://www.sciencedirect.com/science/article/pii/S107599642100069X?via%3Dihub#sec3) | [Prevalence of Antimicrobial Resistance among Clinical Isolates of Bacteroides fragilis Group in Canada in 2010-2011: CANWARD Surveillance Study – PMC](https://pmc.ncbi.nlm.nih.gov/articles/PMC3294939/) | [Guidance: Infection Prevention and Control Measures for Healthcare Workers in All Healthcare Settings – Canada.ca](https://www.canada.ca/en/public-health/services/infectious-diseases/nosocomial-occupational-infections/guidance-infection-prevention-control-measures-healthcare-workers-healthcare-settings.html) |
| Drug-resistant Salmonella spp. (Typhoidal) | [Canada.ca/TyphoidFever](https://www.canada.ca/en/public-health/services/diseases/typhoid-fever/treatment.html) | 2017: [Canadian Integrated Program for Antimicrobial Resistance Surveillance (CIPARS) 2017](https://publications.gc.ca/collections/collection_2019/aspc-phac/HP2-4-2017-4-eng.pdf)  2018: [Canadian Integrated Program for Antimicrobial Resistance Surveillance (CIPARS) 2018](https://publications.gc.ca/collections/collection_2020/aspc-phac/HP2-4-2018-eng-4.pdf)  2019: [Canadian Integrated Program for Antimicrobial Resistance Surveillance (CIPARS) 2019](https://publications.gc.ca/collections/collection_2022/aspc-phac/HP2-4-2019-eng-4.pdf)  2020-21: [Canadian Integrated Program for Antimicrobial Resistance Surveillance (CIPARS)](https://www.canada.ca/content/dam/phac-aspc/documents/services/publications/drugs-health-products-canadian-integrated-program-antimicrobial-resistance-surveillance-2022-executive-summary/canadian-integrated-program-antimicrobial-resistance-surveillance-2022-executive-summary-en.pdf) | [Typhoid vaccines: Canadian Immunization Guide – Canada.ca](https://www.canada.ca/en/public-health/services/publications/healthy-living/canadian-immunization-guide-part-4-active-vaccines/page-23-typhoid-vaccine.html#a2) | [Typhoid vaccines: Canadian Immunization Guide – Canada.ca](https://www.canada.ca/en/public-health/services/publications/healthy-living/canadian-immunization-guide-part-4-active-vaccines/page-23-typhoid-vaccine.html) |
| Extended spectrum B-lactamase-producing Enterobacterales | [German, GJ et al., 2016](https://www.canada.ca/en/public-health/services/reports-publications/canada-communicable-disease-report-ccdr/monthly-issue/2016-42/ccdr-volume-42-4-april-7-2016/ccdr-volume-42-4-april-7-2016-vaccine-preventable-diseases-3.html);  [ISDA, 2024](https://www.idsociety.org/practice-guideline/amr-guidance/#Section1:Extended-spectrum%CE%B2-lactamase-ProducingEnterobacterales);  [Blondeau, J., Charles, M.K., Loo, V. et al., 2023](https://www.nature.com/articles/s41598-023-40012-z#citeas);  [Tamma et al, 2022](https://pmc.ncbi.nlm.nih.gov/articles/PMC9890506/) | [CNSIP 2017-2021 report p.241](https://www.ncbi.nlm.nih.gov/pmc/articles/PMC10903608/pdf/CCDR-49-235.pdf) | [ESBL-positive Escherichia coli and Klebsiella pneumoniae isolates from across Canada: CANWARD surveillance study, 2007–18 \| Journal of Antimicrobial Chemotherapy \| Oxford Academic](https://academic.oup.com/jac/article/76/11/2815/6347675?login=false) | [Guidance: Infection Prevention and Control Measures for Healthcare Workers in All Healthcare Settings – Canada.ca](https://www.canada.ca/en/public-health/services/infectious-diseases/nosocomial-occupational-infections/guidance-infection-prevention-control-measures-healthcare-workers-healthcare-settings.html) |
| Drug-resistant *Campylobacter* spp. | [Gaudreau C, et al., 2016](https://pmc.ncbi.nlm.nih.gov/articles/PMC4994334/) | [CIPARS 2022 executive summary, p.5](https://www.canada.ca/content/dam/phac-aspc/documents/services/publications/drugs-health-products-canadian-integrated-program-antimicrobial-resistance-surveillance-2022-executive-summary/canadian-integrated-program-antimicrobial-resistance-surveillance-2022-executive-summary-en.pdf) | [Multidrug-Resistant Campylobacter coli in Men Who Have Sex with Men, Quebec, Canada, 2015 – PMC](https://pmc.ncbi.nlm.nih.gov/articles/PMC4994334/)  [canadian-integrated-program-antimicrobial-resistance-surveillance-2022-executive-summary-en.pdf](https://www.canada.ca/content/dam/phac-aspc/documents/services/publications/drugs-health-products-canadian-integrated-program-antimicrobial-resistance-surveillance-2022-executive-summary/canadian-integrated-program-antimicrobial-resistance-surveillance-2022-executive-summary-en.pdf)  [For health professionals treating campylobacteriosis (Campylobacter) – Canada.ca](https://www.canada.ca/en/public-health/services/diseases/campylobacteriosis-campylobacter/for-health-professionals.html)  [Antimicrobial Resistance of Human Campylobacter Species Infections in Saskatchewan, Canada (1999-2006): A Historical Provincial Collection of All Reported Cases – PubMed](https://pubmed.ncbi.nlm.nih.gov/31661323/) | [Prevention of campylobacteriosis (Campylobacter) – Canada.ca](https://www.canada.ca/en/public-health/services/diseases/campylobacteriosis-campylobacter/prevention.html) |
| Drug-resistant Pulmonary nontuberculosis *Mycobacteria* | [Canadian Tuberculosis Standard, 7^th^ edition](https://www.canada.ca/content/dam/phac-aspc/migration/phac-aspc/tbpc-latb/pubs/tb-canada-7/assets/pdf/tb-standards-tb-normes-ch11-eng.pdf) | Canadian Tuberculosis Standards 7 t h Edition Chapter 11: Nontuberculous Mycobacteria  <https://www.phac-aspc.gc.ca/tbpc-latb/pubs/tb-canada-7/assets/pdf/tb-standards-tb-normes-ch11-eng.pdf> | Canadian Tuberculosis Standards 7 t h Edition Chapter 11: Nontuberculous Mycobacteria  <https://www.phac-aspc.gc.ca/tbpc-latb/pubs/tb-canada-7/assets/pdf/tb-standards-tb-normes-ch11-eng.pdf> | Canadian Tuberculosis Standards 7 t h Edition Chapter 11: Nontuberculous Mycobacteria  <https://www.phac-aspc.gc.ca/tbpc-latb/pubs/tb-canada-7/assets/pdf/tb-standards-tb-normes-ch11-eng.pdf> |
| Vancomycin-resistant *Enterococcus spp.* | [Levutys et al. 2023;](https://www.ncbi.nlm.nih.gov/books/NBK513233/) [CARSS,2024;](https://www.canada.ca/en/public-health/services/publications/drugs-health-products/canadian-antimicrobial-resistance-surveillance-system-report-2022.html#a2.2) | [CNISP 2017-2021 report p.239](https://www.canada.ca/content/dam/phac-aspc/documents/services/reports-publications/canada-communicable-disease-report-ccdr/monthly-issue/2023-49/issue-5-may-2023/ccdrv49i05a09-eng.pdf) | [CNISP 2017-2021 report p.239](https://www.canada.ca/content/dam/phac-aspc/documents/services/reports-publications/canada-communicable-disease-report-ccdr/monthly-issue/2023-49/issue-5-may-2023/ccdrv49i05a09-eng.pdf) | [Vancomycin-resistant Enterococci (VRE) – Canada.ca](https://www.canada.ca/en/public-health/services/infectious-diseases/nosocomial-occupational-infections/vancomycin-resistant-enterococci.html) |
| Drug-resistant Human immunodeficieny virus | [Canada.ca/PathogenSafetyDataSheet](https://www.canada.ca/en/public-health/services/laboratory-biosafety-biosecurity/pathogen-safety-data-sheets-risk-assessment/human-immunodeficiency-virus.html) | [The Epidemiology of HIV in Canada \| CATIE – Canada’s source for HIV and hepatitis C information](https://www.catie.ca/the-epidemiology-of-hiv-in-canada) | [The Epidemiology of HIV in Canada \| CATIE – Canada’s source for HIV and hepatitis C information](https://www.catie.ca/the-epidemiology-of-hiv-in-canada)  [Drug resistance and resistance testing \| CATIE – Canada’s source for HIV and hepatitis C information;](https://www.catie.ca/your-guide-to-hiv-treatment/drug-resistance-and-resistance-testing)  [HIV in Canada: 2023 surveillance highlights – Canada.ca;](https://www.canada.ca/en/public-health/services/publications/diseases-conditions/hiv-2023-surveillance-highlights-infographic.html)  [The impact of routine HIV drug resistance testing in Ontario: A controlled interrupted time series study – PubMed](https://pubmed.ncbi.nlm.nih.gov/33798201/) | [HIV and AIDS: Prevention and risks – Canada.ca](https://www.canada.ca/en/public-health/services/diseases/hiv-aids/prevention-risks.html) |
| *Mycoplasma genitalium* | [PHAC, 2021;](https://www.canada.ca/en/public-health/services/infectious-diseases/sexual-health-sexually-transmitted-infections/canadian-guidelines/mycoplasma-genitalium/treatment-follow-up.html#treatment2)  [Lê et al, 2023](https://pubmed.ncbi.nlm.nih.gov/38504874/),  NMLB (Internal) – reader must request directly from program area | [Mycoplasma Genitalium: Treatment and follow-up – Canada.ca](https://www.canada.ca/en/public-health/services/infectious-diseases/sexual-health-sexually-transmitted-infections/canadian-guidelines/mycoplasma-genitalium/treatment-follow-up.html#national) | [Lê et al, 2023](https://pubmed.ncbi.nlm.nih.gov/38504874/),  FROM EQUITY REVIEW:  [Researchers study M. genitalium in Montreal \| CATIE – Canada’s source for HIV and hepatitis C information;](https://www.catie.ca/treatmentupdate-251/researchers-study-m-genitalium-in-montreal)  [Mycoplasma Genitalium: Key information and resources – Canada.ca;](https://www.canada.ca/en/public-health/services/infectious-diseases/sexual-health-sexually-transmitted-infections/canadian-guidelines/mycoplasma-genitalium.html) | [Mycoplasma Genitalium: Prevention and control – Canada.ca](https://www.canada.ca/en/public-health/services/infectious-diseases/sexual-health-sexually-transmitted-infections/canadian-guidelines/mycoplasma-genitalium/prevention-control.html) |
| 7Drug-resistant Salmonella spp. (Non-typhoidal) | [CATMAT Interim Guidance](https://www.canada.ca/en/public-health/services/catmat/interim-guidance-management-infections-multidrug-resistant-strain-salmonella-newport.html#a4) | 2017: [Canadian Integrated Program for Antimicrobial Resistance Surveillance (CIPARS) 2017](https://publications.gc.ca/collections/collection_2019/aspc-phac/HP2-4-2017-4-eng.pdf)  2018: [Canadian Integrated Program for Antimicrobial Resistance Surveillance (CIPARS) 2018](https://publications.gc.ca/collections/collection_2020/aspc-phac/HP2-4-2018-eng-4.pdf)  2019: [Canadian Integrated Program for Antimicrobial Resistance Surveillance (CIPARS) 2019](https://publications.gc.ca/collections/collection_2022/aspc-phac/HP2-4-2019-eng-4.pdf)  2020-21: [Canadian Integrated Program for Antimicrobial Resistance Surveillance (CIPARS)](https://www.canada.ca/content/dam/phac-aspc/documents/services/publications/drugs-health-products-canadian-integrated-program-antimicrobial-resistance-surveillance-2022-executive-summary/canadian-integrated-program-antimicrobial-resistance-surveillance-2022-executive-summary-en.pdf) | [nhfi_food_safety_for_first_nations_people_of_canada.pdf](https://www.gov.mb.ca/inr/pdf/pubs/nhfi_food_safety_for_first_nations_people_of_canada.pdf) | [Interim guidance on management of infections with a multidrug-resistant strain of Salmonella Newport - Canada.ca](https://www.canada.ca/en/public-health/services/catmat/interim-guidance-management-infections-multidrug-resistant-strain-salmonella-newport.html) |
| ESBL-Salmonella | [CATMAT Interim Guidance](https://www.canada.ca/en/public-health/services/catmat/interim-guidance-management-infections-multidrug-resistant-strain-salmonella-newport.html#a4) | 2017: [Canadian Integrated Program for Antimicrobial Resistance Surveillance (CIPARS) 2017](https://publications.gc.ca/collections/collection_2019/aspc-phac/HP2-4-2017-4-eng.pdf)  2018: [Canadian Integrated Program for Antimicrobial Resistance Surveillance (CIPARS) 2018](https://publications.gc.ca/collections/collection_2020/aspc-phac/HP2-4-2018-eng-4.pdf)  2019: [Canadian Integrated Program for Antimicrobial Resistance Surveillance (CIPARS) 2019](https://publications.gc.ca/collections/collection_2022/aspc-phac/HP2-4-2019-eng-4.pdf)  2020-21: [Canadian Integrated Program for Antimicrobial Resistance Surveillance (CIPARS)](https://www.canada.ca/content/dam/phac-aspc/documents/services/publications/drugs-health-products-canadian-integrated-program-antimicrobial-resistance-surveillance-2022-executive-summary/canadian-integrated-program-antimicrobial-resistance-surveillance-2022-executive-summary-en.pdf) | [nhfi_food_safety_for_first_nations_people_of_canada.pdf](https://www.gov.mb.ca/inr/pdf/pubs/nhfi_food_safety_for_first_nations_people_of_canada.pdf) | [Interim guidance on management of infections with a multidrug-resistant strain of Salmonella Newport - Canada.ca](https://www.canada.ca/en/public-health/services/catmat/interim-guidance-management-infections-multidrug-resistant-strain-salmonella-newport.html) |
| Drug-resistant *Influenza* A | PHAC FLU WATCH:  [2016-2017](https://www.canada.ca/content/dam/hc-sc/healthy-canadians/migration/publications/diseases-conditions-maladies-affections/fluwatch-2016-2017-51-52-surveillance-influenza/alt/fluwatch-2016-2017-51-52-surveillance-influenza-eng.pdf);  [2017-2018;](https://www.canada.ca/content/dam/phac-aspc/documents/services/publications/diseases-conditions/fluwatch/2017-2018/weeks51-52-december-17-30-2017/weeks51-52-december-17-30-2017.pdf)  [2018-2019](https://www.canada.ca/content/dam/phac-aspc/documents/services/publications/diseases-conditions/fluwatch/2018-2019/week51-52-december-16-december-29-2018/week51-52-december-16-december-29-2018.pdf);  [2019-2020](https://www.canada.ca/content/dam/phac-aspc/documents/services/publications/diseases-conditions/fluwatch/2019-2020/week51/pub-eng.pdf);  [2020-2021;](https://www.canada.ca/content/dam/phac-aspc/documents/services/publications/diseases-conditions/fluwatch/2020-2021/fw-weeks51-53-2021.pdf)  [2021-2022](https://www.canada.ca/content/dam/phac-aspc/documents/services/publications/diseases-conditions/fluwatch/2021-2022/fw-weeks50-52-2021-en.pdf) | PHAC FLU WATCH:  [2016-2017](https://www.canada.ca/content/dam/hc-sc/healthy-canadians/migration/publications/diseases-conditions-maladies-affections/fluwatch-2016-2017-51-52-surveillance-influenza/alt/fluwatch-2016-2017-51-52-surveillance-influenza-eng.pdf);  [2017-2018;](https://www.canada.ca/content/dam/phac-aspc/documents/services/publications/diseases-conditions/fluwatch/2017-2018/weeks51-52-december-17-30-2017/weeks51-52-december-17-30-2017.pdf)  [2018-2019](https://www.canada.ca/content/dam/phac-aspc/documents/services/publications/diseases-conditions/fluwatch/2018-2019/week51-52-december-16-december-29-2018/week51-52-december-16-december-29-2018.pdf);  [2019-2020](https://www.canada.ca/content/dam/phac-aspc/documents/services/publications/diseases-conditions/fluwatch/2019-2020/week51/pub-eng.pdf);  [2020-2021;](https://www.canada.ca/content/dam/phac-aspc/documents/services/publications/diseases-conditions/fluwatch/2020-2021/fw-weeks51-53-2021.pdf)  [2021-2022](https://www.canada.ca/content/dam/phac-aspc/documents/services/publications/diseases-conditions/fluwatch/2021-2022/fw-weeks50-52-2021-en.pdf) | [Surviving influenza: lived experiences of health inequity and pandemic disease in Canada](https://pmc.ncbi.nlm.nih.gov/articles/PMC7828852/pdf/192e688.pdf)  [Influenza: Canadian respiratory virus surveillance report (FluWatch+) — Canada.ca;](https://health-infobase.canada.ca/respiratory-virus-surveillance/influenza.html)  [Influenza A virus subtypes H5, H7, and H9: Infectious substances pathogen safety data sheet - Canada.ca;](https://www.canada.ca/en/public-health/services/laboratory-biosafety-biosecurity/pathogen-safety-data-sheets-risk-assessment/influenza-a-virus-subtypes-h5-h7-h9.html)  [Summary: Canadian respiratory virus surveillance report (FluWatch+) — Canada.ca;](https://health-infobase.canada.ca/respiratory-virus-surveillance/) | [Flu (influenza): Prevention and risks - Canada.ca](https://www.canada.ca/en/public-health/services/diseases/flu-influenza/prevention-risks.html) |
| *Ureaplasma  spp.* | [PHAC, 2008;](https://publications.gc.ca/collections/collection_2011/aspc-phac/HP40-1-2010-eng.pdf) No Canadian data available; [Waites et al., 2005 – No Canadian data available;](https://pmc.ncbi.nlm.nih.gov/articles/PMC1265909/#sec32) | [Case definitions: Nationally notifiable diseases](https://diseases.canada.ca/notifiable/diseases-list) | [Pathogen Safety Data Sheets: Infectious Substances – Ureaplasma urealyticum - Canada.ca](https://www.canada.ca/en/public-health/services/laboratory-biosafety-biosecurity/pathogen-safety-data-sheets-risk-assessment/ureaplasma-urealyticum.html) | [Pathogen Safety Data Sheets: Infectious Substances – Ureaplasma urealyticum - Canada.ca](https://www.canada.ca/en/public-health/services/laboratory-biosafety-biosecurity/pathogen-safety-data-sheets-risk-assessment/ureaplasma-urealyticum.html) |
| *Clostridium difficile* | [Vivian G. et al, 2018](https://utppublishing.com/doi/full/10.3138/jammi.2018.02.13#:~:text=Antimicrobial%20treatment%20options%20are%20metronidazole,including%20metronidazole%20for%20mild%20CDI.);  [MUMs,](https://www.mumshealth.com/online/anti-infective) [Sunnybrook;](https://sunnybrook.ca/content/?page=antimicrobial-stewardship-treatment-guidelines)  [Marchandin H, Anjou C, Poulen G, et al., 2023 (France);](https://pubmed.ncbi.nlm.nih.gov/37352110/)  NMLB (Internal) – reader must request directly from program area | [CNISP 2017-2021 report p.237](https://www.canada.ca/content/dam/phac-aspc/documents/services/reports-publications/canada-communicable-disease-report-ccdr/monthly-issue/2023-49/issue-5-may-2023/ccdrv49i05a09-eng.pdf) | [C. difficile (Clostridium difficile) - Canada.ca](https://www.canada.ca/en/public-health/services/diseases/c-difficile.html) | [C. difficile (Clostridium difficile) - Canada.ca](https://www.canada.ca/en/public-health/services/diseases/c-difficile.html) |
| Drug -resistant *Candida* spp.* | [Bow, et al, 2010;](https://pmc.ncbi.nlm.nih.gov/articles/PMC3009581/)  [CNISP, 2018-2022](https://www.canada.ca/content/dam/phac-aspc/documents/services/reports-publications/canada-communicable-disease-report-ccdr/monthly-issue/2024-50/issue-6-june-2024/ccdrv50i06a03-eng.pdf) | [Case definitions: Nationally notifiable diseases](https://diseases.canada.ca/notifiable/diseases-list) | [Pathogen Safety Data Sheets: Infectious Substances – Candida albicans - Canada.ca](https://www.canada.ca/en/public-health/services/laboratory-biosafety-biosecurity/pathogen-safety-data-sheets-risk-assessment/candida-albicans-pathogen-safety-data-sheet.html) | [Pathogen Safety Data Sheets: Infectious Substances – Candida albicans - Canada.ca](https://www.canada.ca/en/public-health/services/laboratory-biosafety-biosecurity/pathogen-safety-data-sheets-risk-assessment/candida-albicans-pathogen-safety-data-sheet.html) |
| Drug-resistant *Helicobacter pylori* | [Velduyzen van Zanten, 2023;](https://thischangedmypractice.com/management-of-helicobacter-pylori/)  [Krahn et al., 2023](https://academic.oup.com/jcag/article/7/3/221/7473647#463637925) | Helicobacter pylori: Infectious substances pathogen safety data sheet: <https://www.canada.ca/en/public-health/services/laboratory-biosafety-biosecurity/pathogen-safety-data-sheets-risk-assessment/helicobacter-pylori.html> | [Antibiotic dispensation rates among participants in community-driven health research projects in Arctic Canada - PMC](https://pmc.ncbi.nlm.nih.gov/articles/PMC6631451/)  [Antimicrobial susceptibility of Canadian isolates of Helicobacter pylori in Northeastern Ontario - PubMed](https://pubmed.ncbi.nlm.nih.gov/26236355/) | [Helicobacter pylori – National Collaborating Centre for Infectious Diseases](https://nccid.ca/debrief/helicobacter-pylori/) |
| Drug-resistant *Treponema pallidum* | [Canada, Syphilis Guide;](https://www.canada.ca/en/public-health/services/infectious-diseases/sexual-health-sexually-transmitted-infections/canadian-guidelines/syphilis/treatment-follow-up.html#a2) [[Syphilis in Canada: Technical report;](https://www.canada.ca/en/services/health/publications/diseases-conditions/syphilis-epidemiological-report.html) [Martin, I. E., 2009](https://pmc.ncbi.nlm.nih.gov/articles/PMC2691066/) | [Case definitions: Nationally notifiable diseases](https://diseases.canada.ca/notifiable/diseases-list) | [Syphilis guide: Etiology and epidemiology - Canada.ca](https://www.canada.ca/en/public-health/services/infectious-diseases/sexual-health-sexually-transmitted-infections/canadian-guidelines/syphilis/etiology-epidemiology.html) | [Syphilis guide: Prevention and control - Canada.ca](https://www.canada.ca/en/public-health/services/infectious-diseases/sexual-health-sexually-transmitted-infections/canadian-guidelines/syphilis/prevention-control.html) |
| Drug-resistant Chlamydia trachomatis | [Canada, Chlamydia and LGV guide](https://www.canada.ca/en/public-health/services/infectious-diseases/sexual-health-sexually-transmitted-infections/canadian-guidelines/chlamydia-lgv.html) | [Chlamydia and LGV guide: Etiology and epidemiology - Canada.ca](https://www.canada.ca/en/public-health/services/infectious-diseases/sexual-health-sexually-transmitted-infections/canadian-guidelines/chlamydia-lgv/etiology-epidemiology.html) | [Syphilis guide: Etiology and epidemiology - Canada.ca](https://www.canada.ca/en/public-health/services/infectious-diseases/sexual-health-sexually-transmitted-infections/canadian-guidelines/syphilis/etiology-epidemiology.html) | [Chlamydia and LGV guide: Prevention and control - Canada.ca](https://www.canada.ca/en/public-health/services/infectious-diseases/sexual-health-sexually-transmitted-infections/canadian-guidelines/chlamydia-lgv/prevention-control.html) |
