# Supplementary material for: Canada’s 2025 AMR priority pathogens: Evidence-based ranking and public health implications
Source: PLoS One. 2025 Sep 17;20(9):e0330128. doi: 10.1371/journal.pone.0330128 (PMC12443280; doi:10.1371/journal.pone.0330128)
Supplement: S2 File — (DOCX) [file pone.0330128.s002.docx]

**S2. Quality of Evidence: Evaluation of Sources used to inform Multi-Criteria Pathogen Scoring**

| **Quality of Evidence** | **% (proportion)** | **Data Sources*** |
| --- | --- | --- |
| **Very Good** | 45% (13/29) | ARNI, AMRNet, CBTLSS, CIPARS, CNISP, ESAG, eStrep, and FoodNet Canada, GASP, PHAC STI guidelines, PHAC Pathogen safety Sheets, Peer-reviewed studies, P/T surveillance, MUMS |
| **Good** | 48% (14/29) | May include data sources from “Very Good” but heavily relied on P/T surveillance systems , and Canadian peer-reviewed target studies |
| **Fair** | 7% (2/29) | Includes limited Canadian and P/T data and populated with International surveillance systems/studies with comparable health infrastructure to Canada |

**Scoring: Quality of Evidence**

| AMR Pathogen | Quality of Evidence | | | | | | | | |
| --- | --- | --- | --- | --- | --- | --- | --- | --- | --- |
|  | 1  ( Scoring options) | 2 | 3 | 4 | 5 | 6 | 7 | 8 | 9 |
| *Candida auris* | Very good | Very good | Very good | Good | Very good | Fair | Fair | Fair | Fair |
| Carbapenem-resistant Enterobacterales | Very good | Very good | Very good | Good | Very good | Good | Fair | Fair | Fair |
| Drug-resistant *Neisseria gonorrhoeae* | Very good | Very good | Very good | Very good | Very good | Fair | Fair | Fair | Fair |
| Drug-resistant Invasive Group A Streptococcus | Very good | Very good | Very good | Fair | Very good | Fair | Fair | Fair | Fair |
| Drug-resistant Streptococcus pneumoniae | Very good | Very good | Very good | Fair | Very good | Fair | Fair | Fair | Fair |
| Drug-resistant *Aspergillus* spp. | Good | Good | Good | Fair | Good | Good | Good | Good | Very good |
| Multi-drug resistant Mycobacterium tuberculosis | Very good | Very good | Very good | Very good | Very good | Fair | Fair | Fair | Fair |
| Drug-resistant *Shigella* spp. | Good | Good | Good | Good | Good | Good | Good | Good | Fair |
| Drug-resistant *Haemophilus influenzae* | Good | Good | Very good | Fair | Very good | Fair | Good | Good | Fair |
| Carbapenem-resistant *Pseudomonas aeruginosa* | Good | Good | Good | Fair | Good | Good | Good | Good | Fair |
| Methicillin-Resistant *Staphylococcus aureus* | Very good | Very good | Very good | Very good | Very good | Good | Fair | Fair | Fair |
| Drug-resistant Invasive Group B Streptococcus | Very good | Very good | Very good | Fair | Very good | Fair | Fair | Good | Fair |
| Carbapenem-resistant *Acinetobacter spp.* | Very good | Very good | Very good | Fair | Fair | Fair | Fair | Fair | Fair |
| Drug-resistant *Bacteroides* spp. | Fair | Fair | Good | Fair | Good | Good | Good | Very good | Fair |
| Drug-resistant Salmonella spp. (Typhoidal) | Very good | Very good | Very good | Fair | Very good | Fair | Fair | Good | Fair |
| Extended spectrum B-lactamase-producing Enterobacterales | Very good |  | Very good | Fair | Good | Good | Fair | Good | Fair |
| Drug-resistant *Campylobacter* spp. | Very good | Very good | Very good | Fair | Very good | Good | Fair | Good | Fair |
| Drug-resistant Pulmonary nontuberculosis *Mycobacteria* | Fair | Fair | Very good | Very good | Very good | Fair | Fair | Fair | Fair |
| Vancomycin-resistant *Enterococcus spp.* | Very good | Very good | Very good | Very good | Very good | Good | Fair | Fair | Fair |
| Drug-resistant Human immunodeficiency virus | Fair | Fair | Good | Fair | Very good | Fair | Fair | Fair | Fair |
| *Mycoplasma genitalium* | Good | Good | Very good | Good | Very good | Good | Fair | Good | Fair |
| Drug-resistant Salmonella spp. (Non-typhoidal) | Very good | Very good | Very good | Fair | Very good | Fair | Fair | Good | Fair |
| ESBL-Salmonella | Very good | Very good | Very good | Fair | Very good | Fair | Fair | Good | Fair |
| Drug-resistant *Influenza* A | Very good | Very good | Very good | Fair | Very good | Fair | Fair | Fair | Fair |
| *Ureaplasma  spp.* | Good | Good | Fair | Fair | Fair | Very good | Good | Fair | Very good |
| *Clostridium difficile* | Very good | Very good | Very good | Very good | Very good | Fair | Fair | Fair | Fair |
| Drug -resistant *Candida* spp.* | Fair | Fair | Very good | Fair | Very good | Good | Fair | Good | Good |
| Drug-resistant *Helicobacter pylori* | Good | Good | Very good | Fair | Very good | Good | Good | Good | Good |
| Drug-resistant *Treponema pallidum* | Fair | Fair | Very good | Fair | Good | Very good | Fair | Fair | Fair |
| Drug-resistant Chlamydia trachomatis | Very good | Very good | Very good | Fair | Very good | Fair | Fair | Fair | Fair |
